# Extreme Magneto-transport of Bulk Carbon Nanotubes in Sorted Electronic Concentrations and Aligned High Performance Fiber

**DOI:** 10.1038/s41598-017-12546-6

**Published:** 2017-09-22

**Authors:** John S. Bulmer, Agnieszka Lekawa-Raus, Dwight G. Rickel, Fedor F. Balakirev, Krzysztof K. Koziol

**Affiliations:** 1 Department of Materials Science and Metallurgy, University of Cambridge, 27 Charles Babbage Rd., Cambridge, UK; 2 Faculty of Mechatronics, Warsaw University of Technology, Warsaw, PL Poland; 3 National High Magnetic Field Laboratory, Pulsed Field Facility, Bldg. 127, Los Alamos, New Mexico 87545 USA; 4 Cranfield University, School of Aerospace, Transport and Manufacturing, Cranfield, Bedfordshire MK43 0AL, United Kingdom

## Abstract

We explored high-field (60 T) magneto-resistance (MR) with two carbon nanotube (CNT) material classes: (1) unaligned single-wall CNTs (SWCNT) films with controlled metallic SWCNT concentrations and doping degree and (2) CNT fiber with aligned, long-length microstructure. All unaligned SWCNT films showed localized hopping transport where high-field MR saturation definitively supports spin polarization instead of a more prevalent wave function shrinking mechanism. Nitric acid exposure induced an insulator to metal transition and reduced the positive MR component. Aligned CNT fiber, already on the metal side of the insulator to metal transition, had positive MR without saturation and was assigned to classical MR involving electronic mobility. Subtracting high-field fits from the aligned fiber’s MR yielded an unconfounded negative MR, which was assigned to weak localization. It is concluded that fluctuation induced tunnelling, an extrinsic transport model accounting for most of the aligned fiber’s room temperature resistance, appears to lack MR field dependence.

## Introduction

Potentially competing with conductive metals for weight-critical electromagnetic applications^1^, bulk textiles composed of single wall carbon nanotubes (SWCNT) are gradually increasing in strength, thermal and electrical conductivity. In part this gradual improvement is due to incremental enhancement of microstructure alignment, doping, graphitic crystallinity, and metallic SWCNT concentration, although sometimes gradual enhancement leads to a discrete step in SWCNTs textile performance. In one magneto-transport study for example^2^, unaligned SWCNT films have a metallic SWCNT concentration threshold (~75%) below which there is inefficient hopping transport with localized charge carriers and a resistance that diverges approaching absolute zero. Their response to a magnetic field is initially negative magneto-resistance (MR) followed by a turn to positive MR. With a concentration greater than 75%, a discrete insulator to metal transition occurs where charge carriers are delocalized, resistance remains finite approaching absolute zero, and there is only a negative MR response with field (at least to their magnet limit, 6 T). With their particular experiment however, vacuum baking prior to measurement removed physisorbed oxygen and de-doped the SWCNTs. Real world, practical SWCNT conductors however will likely have atmospheric exposure and possibly deliberate chemical species intercalation. In this chemically treated state, counterintuitively, it has been shown that semi-conducting unaligned SWCNT films are more conductive than metallic SWCNT films, chemically treated or otherwise^3,4^. Discrete insulator to metal transitions, similar to the metallic SWCNT threshold, have also been observed with controlling the continuous degree of chemical doping^5,6^. These doping studies too have resulted in a suppression of the positive MR component. The importance of metallic SWCNT concentration in a chemically treated state to bulk transport is possibly under question, as well as the extent of suppression of the positive MR component in metallic or doped SWCNT materials.

In addition to metallic species concentration and doping, graphitic crystallinity is also important to the overall bulk electric transport. There is a strong correlation between high conductivity and high graphitic crystallinity in CNT fibers^7–10^ as well as older graphitic intercalation compounds^11,12^. The most conductive graphitic intercalation compounds, in some cases surpassing the conductivity of copper at room temperature, had a host graphitic structure with a graphitic crystallinity beyond the resolution capability of X-ray diffraction and Raman spectroscopy. High magnetic field became the best probe for crystallinity in these host graphitic materials^13^. In^14^ we proposed that SWCNT based textiles may also approach this degree of graphitic crystallinity, where Raman spectroscopy looses resolution and other techniques, such as high magnetic field characterization, may become necessary for quality control.

In this report we explore the high-field magneto-transport of unaligned, purified SWCNT film with controlled metallic SWCNT concentration in both the “as-is” and chemically treated/doped states. These commercially obtained, unaligned films have controlled metallic SWCNT concentration (95% metallic, 98% semi-conducting, and unsorted) and known CNT length distribution. At high-field we uncover a saturating positive MR component that indicates a spin polarization mechanisms over a more prevalent shrinking wave function mechanism. With better understanding of these unaligned films, we next explore the high-field transport of bulk CNT fiber with an aligned microstructure. While the aligned CNT fiber, manufactured in-house, does not have the high purity or controlled electronic species concentration, they comprise of internally aligned CNTs that are individually hundreds of times longer and are possible contenders for replacing copper in practical conductive cables^1^. We will find that the high magnetic field is a good probe for graphitic crystallinity in these aligned CNT materials.

We probe magneto-transport by measuring the relative resistance change as a function of temperature (room temperature to 1 K) and magnetic field (0 to 60 T). The temperature dependence of resistance of carbon based materials generally may show various electronic transport mechanisms that are present in the material, the particular side of the insulator to metal transition, as well as determine the split between intrinsic and extrinsic transport^5,6,15,16^. Complementing temperature dependence, magnetic field dependence is generally used to assign values to the parameters in transport models^5,6^ can resolve ambiguity between transport mechanisms^17–20^ and is a probe for electronic mobility and graphitic crystallinity^13^. We will show that the relatively high magnetic fields we use (60 T) are necessary to unambiguously assign magneto-transport mechanisms across the wide temperature range considered. The MR mechanism selected must be consistent with the transport mechanism indicated by zero-field, temperature dependent resistance measurement^15^ and, for this reason, zero-field results are given before their field dependent results.

## Experimental Set-up

### Unaligned SWCNT film

**Figure 1:**
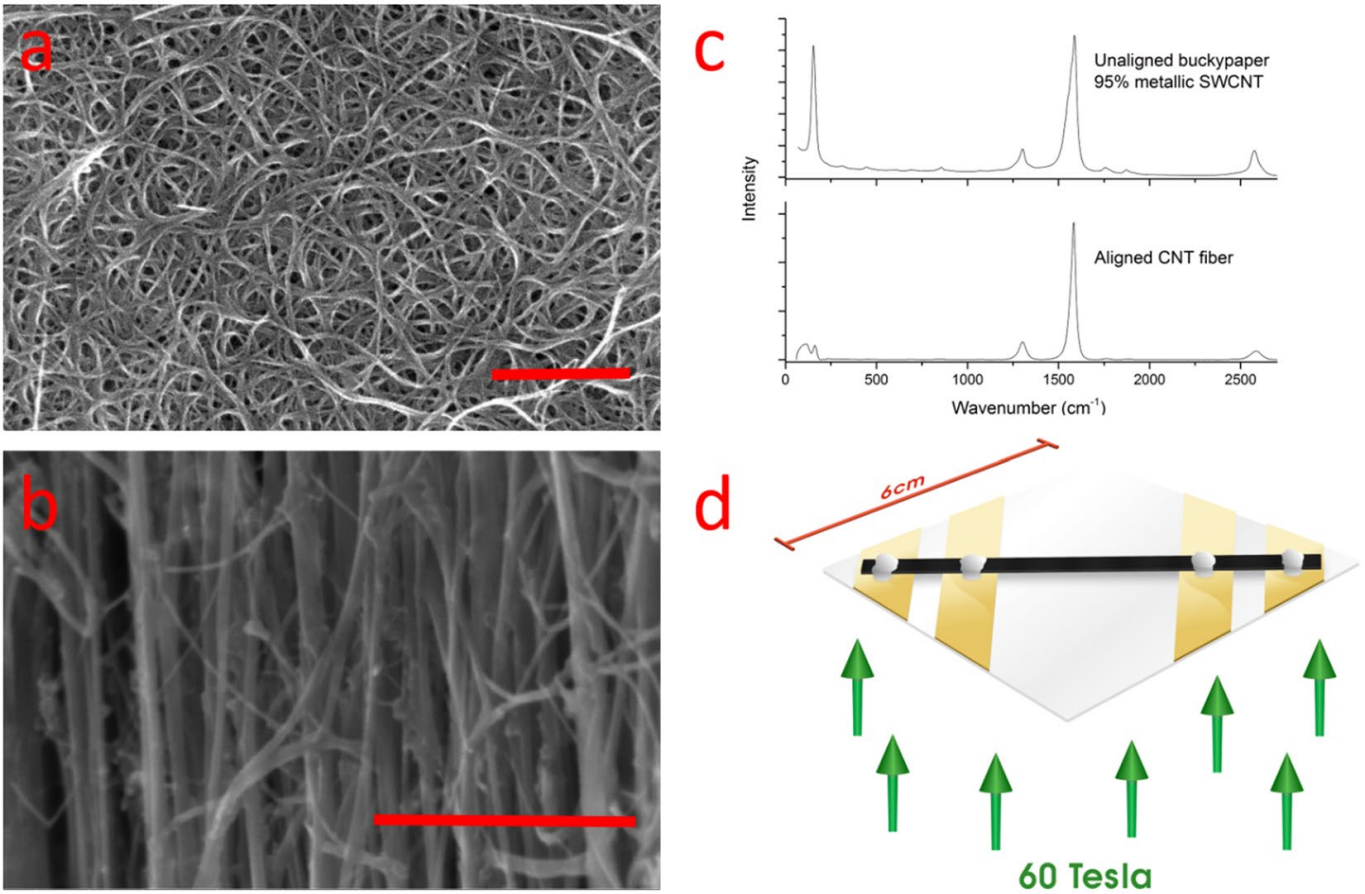
(**a**) Scanning electron photographs of (**a**) unaligned SWCNT buckypaper and (**b**) aligned CNT fiber from in-house floating catalyst chemical vapor deposition. The typical bundle size for the aligned fiber is approximately 30 nm. Red bars indicate 500 nm (**c**) Typical Raman spectra for these materials at a 785 nm laser excitation. Radial breathing modes appearing below 200 cm^−1^ indicate the presence of few-walled CNTs. (**d**) Schematic of the four probe configuration where the field is perpendicular to the probing current flow.

Unaligned SWCNT films obtained from *NanoIntegris* were synthesized by the arc discharge process and then sorted for metallic SWCNT concentration using density gradient centrifugation^21^. This study primarily focused on unaligned films with a metallic SWCNT concentration of 95% and the typical unsorted metallic concentration of 33%, although limited transport measurements were accomplished with a 98% semi-conducting SWCNT film. After sorting and purification, the SWCNTs were filtered through a membrane yielding self-supporting, opaque, unaligned thin films, or buckypaper. Company provided data indicates residual catalyst is less than 1% and carbonaceous impurity is less than 5%, all by weight. Company provided data show that the average SWCNT length is 0.7 µm (metallic SWCNTs) and 1 µm (semi-conducting SWCNTs); diameters range from 1.2 to 1.7 nm, with the average being 1.4 nm. Scanning electron microscopy (Fig. 1a) shows the unaligned CNT bundle microstructure. In-house Renshaw Raman spectroscopy (wavelength 785 nm) shows a G:D ratio of ~15 and prominent radial breathing modes, indicating the presence of SWCNT or few-walled CNTs (Fig. 1c). Further characterization on these samples in particular, to include additional Raman spectroscopy, length distribution, and electron microscopy, may be found in^14^. Further characterization data on these films generally, to include thermogravimetric analysis and UV-vis absorption spectroscopy, is company provided^22^. Note that the drastic difference between films in the zero-field resistance versus temperature plots (to be shown later) qualitatively confirm the sorting process between unsorted, predominantly semi-conducting SWCNTs, and predominantly metallic SWCNTs.

As-is samples, taken after initial transport measurements, were next chemically treated with 70% nitric acid, a well-established CNT dopant^15,23,24^. This was accomplished by carefully transferring a nitric acid drop via pipette to the film and allowing it to dry under a heat lamp until the resistance stabilized, taking approximately an hour. While there is a large amount of residual acid with this technique, ionic conduction processes will be frozen out at low temperature^25^. Transport measurements were then conducted again in the doped state.

### Aligned CNT fiber

Aligned CNT fiber was obtained in an in-house floating catalyst chemical vapor deposition reactor that generates aligned CNT textiles in one single production step. The process is thoroughly covered in the literature^26^. Here, ethanol (the primary carbon source), ferrocene (the catalyst), and thiophene (the promoter) are carried by hydrogen gas into a 1300 °C tube furnace. The resultant catalytic decomposition renders iron catalyst nano-particles saturated with carbon, which results in CNT growth in an unsupported gas phase. The CNT “cloud” is directly pulled out of the reactor and condensed with acetone, rendering a bulk CNT fiber after evaporation. This process results in bulk fiber comprising of predominantly aligned CNT bundles, each CNT commonly said to be about 1 mm long^26^. Residual catalyst and carbonaceous impurity vary between batches, but is typically between 1–15% and 5–15% by weight respectively. Specific conductivity was 345 Sm^2^ kg^−1^ and the conductivity was 200 k Sm^−1^. Scanning electron microscopy shows aligned CNT bundles, with varying diameter though averaging approximately 30 µm (Fig. 1b). Raman spectroscopy yields G:D ratio of ~7 and radial breathing modes indicating the presence of few-walled CNTs (Fig. 1c). Further characterization on this sample in particular may be found in^26^.

### High-Field Magnet

The high-field MR experiments were carried out at the National High Field Magnetic Laboratory in Los Alamos, New Mexico. Magnet time was in high demand, although several separate measurements were made on different pieces of the unaligned SWCNT films and CNT fiber. The results that follow are representative of that larger data set. The resistance *R* of the CNT samples were measured as a function of applied magnetic field *H* and temperature *T*. Samples were mounted in the standard four probe configuration with silver paint onto gold pads supported by sapphire substrate. The length between the inner conductors was 3 mm. The aligned CNT fiber needed to be constrained because electromagnetic induced mechanical vibration under field skewed the MR results. This was accomplished by sandwiching it between the sapphire substrate and another fixed sapphire slab.

The sample, a temperature probe, and a calibrated pick-up coil for field measurement, were housed together in a multi-chamber removable cryostat. The cryostat is placed in a second level, liquid helium refrigerator in the bore of the magnet with the sample aligned to the magnet’s center. Samples were oriented such that the field, when triggered, is perpendicular to the current flow. In the case of the aligned CNT fiber, the current flow was in the direction of the CNT microstructure alignment (Fig. 1d).

As the sample cooled, the DC resistance was measured as a function of temperature at zero-field using standard equipment. Probe current for the DC measurement was typically 10 µA. When a desired temperature is reached and stabilized, the pulse magnet is fired to measure resistance as a function of field. The high-field pulse had a maximum field of 60 T with a 30 ms duration and a 9 ms rise time. To mitigate electromagnetic noise from the magnet, a custom built AC lock-in amplifier technique measured the resistance in conjunction with the pulsed field. A 28 kHz sinusoid signal continually fed into a high impedance circuit containing an isolation transformer and the sample. Current compliance was better than 0.05% and the typical current magnitude through the sample was 100 µA. Using lower probing currents was at the expense of signal to noise ratio, although repeatability with lower current demonstrated that sample heating was not an issue. Current and voltage signals levels were augmented by sensitive operational amplifiers. The 28 kHz signal was well above the magnet’s electromagnetic noise and post process band filtering separated out the MR signal.

A limited amount of transport experiments were performed on 98% semi-conducting SWCNT films where, at cryogenic temperatures, resistance was orders of magnitude greater than other samples and these AC techniques could not be used. In this circumstance, the samples were placed in a separate “long pulse” magnet of 60 T lasting approximately 2.5 s with a 1.2 s rise time. This lowered the electromagnetic shot noise and permitted use of simple DC measurement. The measurement circuit containing the sample was self-contained with an internal battery power supply to prevent alternate paths to ground.

## Results and Discussion–Unaligned SWCNT film

### Unaligned SWCNT Zero-Field Transport

**Figure 2:**
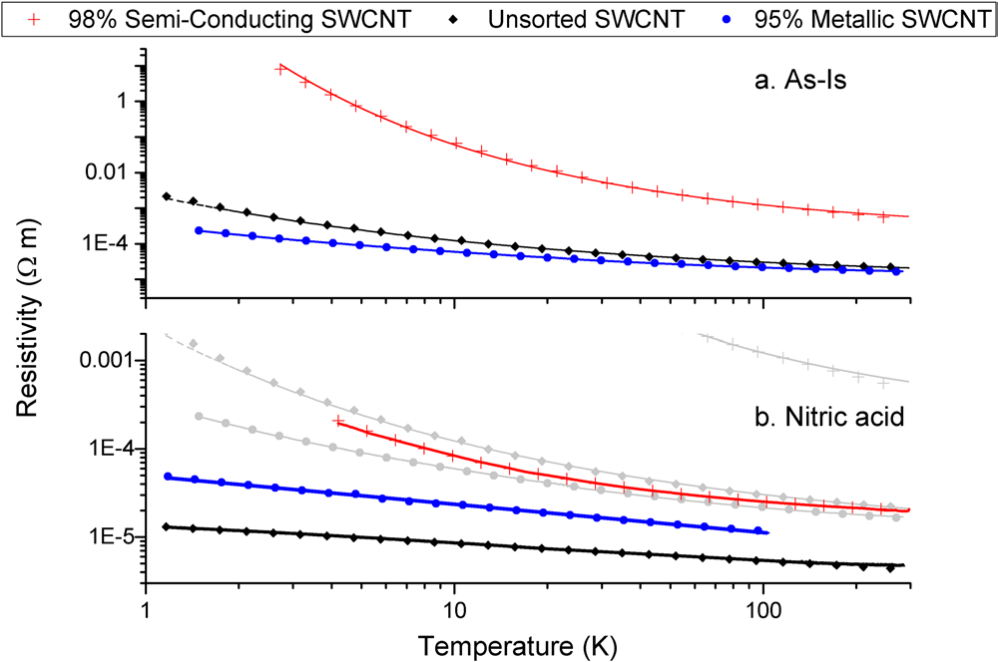
Resistance *R* versus temperature *T* plots for the unaligned SWCNT film in (**a**) the as-is state and (**b**) after nitric acid exposure, a typical doping technique. Points are the data and traces are the best fit models. The as-is samples show hopping conduction for all three metallic SWCNT concentrations with a diverging resistance as absolute zero is approached. Nitric acid treatment induces an insulator to metal transition in the metallic and unsorted samples as indicated by the linearity on the log-log plot. The semi-conducting sample still shows hopping behavior after doping. So that as-is films may be directly compared to acid treated films on the same scale, greyed out traces below represent the as-is material.

We first discuss the results of the unaligned SWCNT films, starting with zero-field *R*/*T* measurement to determine the applicable transport regime before the analysis of magneto-resistance (MR). Figure 2 shows zero-field *R* versus *T* measurements of the “as-is” and chemically treated unaligned SWCNT films. In all cases *R* increases with decreasing *T* (*dR*/*dT* < 0) without any appearance of a metallic-like temperature dependence. This semi-conducting temperature response is present despite the metallic SWCNT concentration and illustrates the overall dominating influence of extrinsic factors, such as CNT junctions, over the intrinsic SWCNT conductivity. For the as-is films, as *T* approaches zero, *R* increases at an ever increasing rate. This suggests charge carrier localization and transport regulated by variable range hopping (VRH). As shown in supplemental information (Figs S1–1 to S1–3) and consistent with previously reported results^2,3^, the semi-conducting SWCNT sample fits best to Efros-Shklovskii (ES) hopping, the unsorted SWCNT sample fits best to two dimensional hopping, and the metallic SWCNT sample films fits best to three dimensional hopping. We point out that in^2^, a metallic SWCNT concentration above 75% resulted in an insulator to metal transition with the *R*/*T* relationship governed by a power law. We did not observe this exact transition in our predominantly metallic as-is SWCNT film, although the general trend in^2^ and our results both indicate an increase in hopping dimension with greater metallic SWCNT concentration. Beyond the differences with atmospheric exposure, there can be many material differences between our predominantly metallic films and those found in^2^ to include: CNT length and diameter, bundle diameter, packing density, and trace impurities left from the sorting process.

After nitric acid treatment, however, overall conductivity for all three films improve and, at least for the metallic and unsorted samples, *R*/*T* now has a power law dependence (indicated by a straight line on the log-log plot) and signals an approach to an insulator to metal transition^6^. The power law exponents are −0.32 (metallic) and −0.2 (unsorted). We note that in some reported cases other SWCNT mats and ropes have also displayed power law behavior with power law exponents ranging from −0.26 to −0.446. This was attributed to Tomonaga–Luttinger-liquid behavior where charge carrier interaction in one dimensional conductors becomes significant relative to extrinsic junction transport^27^. The nitric acid also enhances the conductivity of the semi-conducting sample, although there still appears to be diverging hopping behavior approaching absolute zero.

In the doped state the most conductive sample was not the predominantly metallic SWCNT film. Similar results have been observed before^3,4,28^ where films composed of doped metallic SWCNTs are less conductive than films composed of doped semi-conducting SWCNTs. This was primarily attributed to metallic SWCNT’s greater Fermi level shift required to reach additional conduction bands, compared to the smaller shift required for reaching a semi-conducting SWCNT’s first conduction band. While literature establishes that high metallic SWCNTs concentrations do not necessarily lead to the highest doped bulk conductivities, we also point out that, particular to our situation, on average the metallic SWCNTs are shorter (~0.7 µm compared to ~1 µm for semi-conducting SWCNTs). In addition, the unsorted SWCNTs have not undergone the centrifugation and separation procedures, which could also possibly impact the intrinsic conductivity. Nitric acid doping of the predominantly metallic SWCNT films still led to a significant conductivity improvement however and illustrates the role of chemical treatment on extrinsic junction modification^5,28^.

### High-Field Transport of Unaligned SWCNT Films

**Figure 3:**
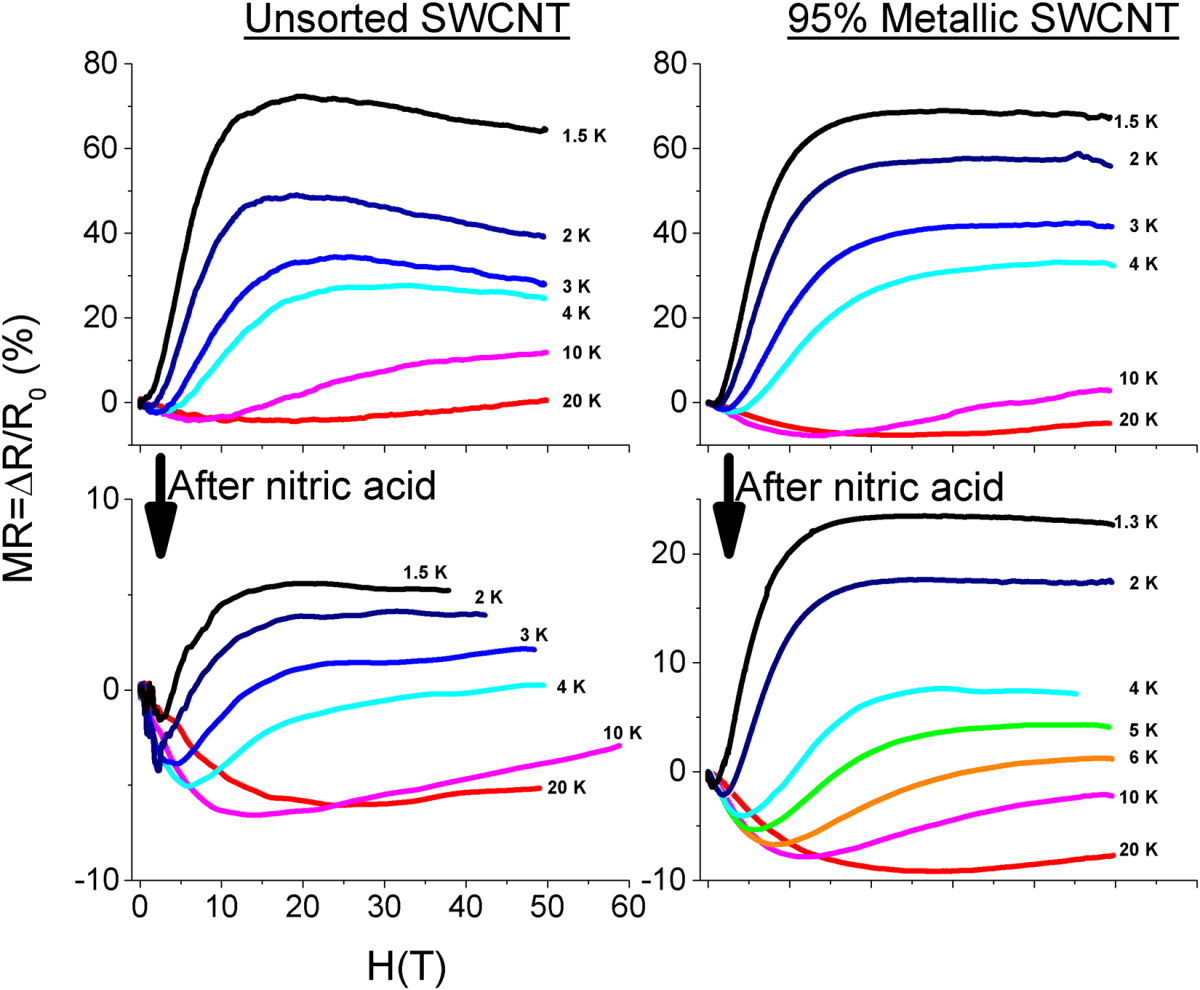
MR as a function of field *H* of the unsorted and metallic SWCNT films, in both the as-is and chemically treated state for a variety of temperatures from ~1.5 to 20 K. *ΔR* = *R(H)* − *R*_0_, where *R*_0_ is the resistance at zero-field. All unaligned films experience a saturation in the positive MR component above 10 T, while acid treatment substantially reduced the magnitude of the positive MR component.

Now that hopping conduction is established for the as-is unaligned material and an approach to an insulator to metal transition is established after nitric acid exposure, Fig. 3 shows the MR response of these films as a function of field *H*, where MR = (*R*(*H*) − *R*(0))/*R*(0). Common to all these samples, for 10 T and below, there is first a negative MR and this is followed by a turn to positive MR at higher field. This positive MR component gets stronger for lower temperatures. This is well documented MR behaviour in CNT materials in general^5,6,23^. Strikingly however, in all samples independent of metallic SWCNT concentration, positive MR is followed by MR saturation at high-field. Note that had the study been restricted to typical fields found in cryogenic laboratories (~10 T), the saturation would not be obvious. Between the as-is metallic and unsorted unaligned SWCNT films, there is not an appreciable difference in MR magnitude at the saturation level. Particular to the as-is unsorted film however, there was a secondary negative MR after the high-field MR level-off. Nitric acid treatment significantly suppresses the positive MR component for both metallic and unsorted SWCNT films, although this chemical induced suppression is more pronounced in the unsorted.

While there are several mechanisms leading to positive MR, the zero-field resistance versus temperature response limits consideration to positive MR mechanisms that fall under the variable range hopping model^15^. There are two prevalent mechanisms of positive MR available to the variable range hopping model, which have both been used in disordered bulk CNT transport: (1) magnetic field induced shrinking of the localized charge carrier wave function and (2) saturation from spin polarization. The more widespread model of the two is the magnetic field induced shrinking of the localized charge carrier wave function^5,16,29–32^. This mechanism turns the spherical wave function distribution of the localized charge carrier into a cigar shape aligned with the field. This means less overlap of the wave function between localized states and an increase in *R* with field *H*, or positive MR. Here, in the low-field limit:

1 $$MR=\exp [{K}_{1}{H}^{2}]-1$$

where *K*_*1*_ ∝ *T* ^*S1*^ is a temperature dependent factor such that *S*1 = −¾ for three dimensional hopping^33^, −1 for two dimensional hopping^29^, and −3/2 for ES hopping^16^. In the high-field limit, at least for three dimensional variable range hopping, positive MR still grows with increasing field according to

2 $$MR+1\propto \exp [{K}_{2}{H}^{1/3}]$$

where *K*_*2*_ ∝ *T* ^*S2*^ and *S*2 = −1/3^33^.

The other positive MR mechanism under variable range hopping, though less prevalent, is MR saturation from spin polarization^6,16,31^. Two localized charge carriers may share a hopping site provided their spins cancel. Field tends to align the charge carrier spin, making pairing at a hopping site less likely and this increases resistance with field. Eventually all of the spins will align and further increasing of field has no effect, resulting in a level off in MR. No closed form expression exists for the full MR response with field and calculation requires numerically solving an involved polynomial equation^34^. Analytical expressions, however, do exist for the particular value of MR level off, *MR*_*SAT*_:

3 $$M{R}_{SAT}=\exp [{K}_{3}{T}^{S3}]-1$$

where *K*_*3*_ is a constant and *S*3 = −1/4 for three dimensional hopping^34^ and −1/3 for two dimensional hopping^35^. The field value where *MR*_*SAT*_ occurs, *H*_*SAT*_, is:

4 $${H}_{SAT}=\exp [{K}_{4}{T}^{S4}]$$

where *K*_*4*_ is a constant and *S4* = 3/4 for three dimensional hopping^34^, 2/3 for two dimensional hopping^35^.

Complicating analysis, both positive MR mechanisms at the low-field limit have MR ∝ *H* ^2^ and it is possible they contribute simultaneously. Indeed, this possibility was explored in low-field magneto-transport studies in CNTs^36,37^ and other disordered materials^38^. We emphasise the qualitative MR level-off in our high-field data for the first time decisively supports the spin saturation model by itself over any contribution from the shrinking wave function model, which expects a continued MR increase with field. Now we fit the spin saturation model to the data to quantitatively consider the model’s fitted parameters.

### High-Field Analysis

**Figure 4:**
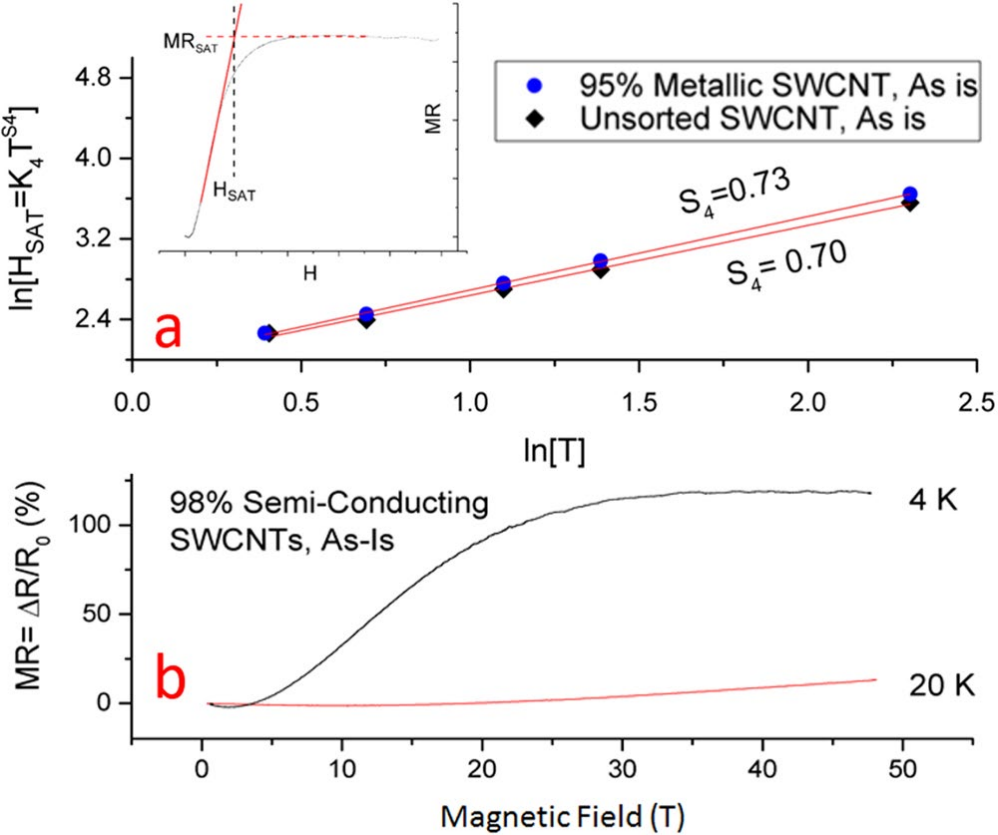
(**a**) For the as-is metallic and unsorted films, log-log plot of temperature *T* against the field where MR saturation occurs *H*_*SAT*_. The power law behavior matches the theoretical values from the spin polarization mechanism. Inset, an example of how the point of saturation, *MR*_*SAT*_ and *H*_*SAT*_, was determined from the MR data tangent lines. (**b**) High-field MR study on the semi-conducting as-is unaligned SWCNT film as a function of field *H*.

Figure 4a plots the field value in which MR level-off occurs, *H*_*SAT*_, against temperature *T* for the as-is unaligned SWCNT films. This point was determined by intersecting tangent lines from the positive MR rise and positive MR saturation as indicated in the inset of Fig. 4. The saturation field’s linearity of the log-log plot with *T* shows a power law response. For the metallic SWCNT film, the exponent was 0.73 and the spin saturation model for its hopping (in three dimensions) expects an exponent of 3/4^34^. For the unsorted SWCNT film, the exponent was 0.70 and the spin saturation model for its hopping (in two dimensions) expects an exponent of 2/3^35^. Thus, these power law exponents are consistent with the spin saturation model.

Note that the MR traces at lower field initially show negative MR and this is from a separate mechanism under variable range hopping^16,39,40^ not related to either the field induced shrinking wave function or saturation from spin polarization. This separate negative MR mechanism would confound attempts fitting the MR data to solely positive MR models. This negative MR is known to become more significant at lower temperatures and will saturate to a field-independent, steady-state value at relatively low-fields^41–43^ anywhere from 0.6 to 2.5 T for the temperature range 4–120 K^32^. Provided the negative contribution indeed saturates and quits changing with field, the high-field value where the observed positive MR level-off occurs, *H*_*SAT*_, should be unaffected by the saturated negative MR mechanism. On the other hand, the MR value of the level-off, *MR*_*SAT*_, is displaced by the negative MR contribution and there can be no expectation that the measured *MR*_*SAT*_ adheres purely to the positive MR model. Indeed this was found to be the case where the relation between the measured *MR*_*SAT*_ and *T* did not follow the expected power law from equation (3) as shown in the supplemental section (Figs S2–1). As also shown in the supplemental information (Figs S3–1), we also fitted the more prevalent shrinking wave function model to the MR data, as demonstrated in^5,30^, and it results in unrealistic fitted parameters. We stress that the level-off in the positive MR qualitatively supports the spin saturation model as the sole source of positive MR. If the wave function shrinking mechanism were contributing at all, we would expect the MR to continually increase with field.

### MR of Chemically Treated Films

As shown earlier in Fig. 3, the qualitative shape of the acid treated MR response is largely similar to the as-is material; that is, there is initially negative MR, followed by positive MR, then MR saturation. The primary difference after acid treatment was the substantial magnitude reduction of the positive MR component. Suppression of the positive MR component has been observed before with other doped or metallic CNT magneto-transport studies in low-field^5,30^ and has been alluded to increased localization length and carrier density under the shrinking wave function model^5^. Under the alternate and better suited spin polarization model used here, a smaller *MR*_*SAT*_ magnitude is also related to larger localization lengths and higher carrier density^34^. As shown earlier in Fig. 2, the acid treated samples’ linearity in the zero-field *R*/*T* log-log plots indicate an approach to the insulator to metal transition. The spin saturation mechanism however applies for three dimensional^34^ and two dimensional^35^ hopping and it is not known how this model applies near the insulator to metal transition. Regardless, Figs S4–1 in the supplemental section plots the measured field saturation values *H*_SAT_ of the chemically treated samples against *T*. A power law fit yields temperature exponents 0.76 and 1.0 for the acid treated metallic and unsorted unaligned SWCNT films respectively.

### Secondary Negative MR

The as-is unsorted film experienced a slight, secondary negative MR after the MR level-off in high-field, which was repeatable across multiple samples (Fig. 3). Note the secondary negative MR is not present after chemical treatment nor is it there for the metallic unaligned SWCNT films. At first glance this secondary negative MR might stem from the contribution of un-doped semi-conducting SWCNTs and this possibility is now discussed. We conducted a limited number of magneto-transport experiments on an unaligned 98% semi-conducting SWCNT film. The material is very resistive at cryogenic temperatures, which ruled out the AC lock-in amplified approach and pulse magnets. Instead, the DC resistance was measured in the “long pulse” magnet, which is resource expensive and permitted only a few long pulse shots. Figure 4b shows the MR for the as-is 98% semi-conducting film at 4 and 20 K. As shown, the positive MR at 4 K saturates at a value four times higher than the unsorted and metallic unaligned SWCNT films at 4 K. The MR saturation, however, stays constant and the secondary negative MR component never develops. This suggests that the secondary negative MR component present on the unsorted, as-is unaligned SWCNT films is not from the semi-conducting SWCNTs themselves. Appearance of a secondary, linear negative MR has been observed in at least one CNT study that measured up to 14 T, although there were no conclusions regarding its origin^44^. This effect needs to be investigated in future high-field studies.

## Results and Discussion— Aligned CNT Fiber

### Zero-Field Transport

**Figure 5:**
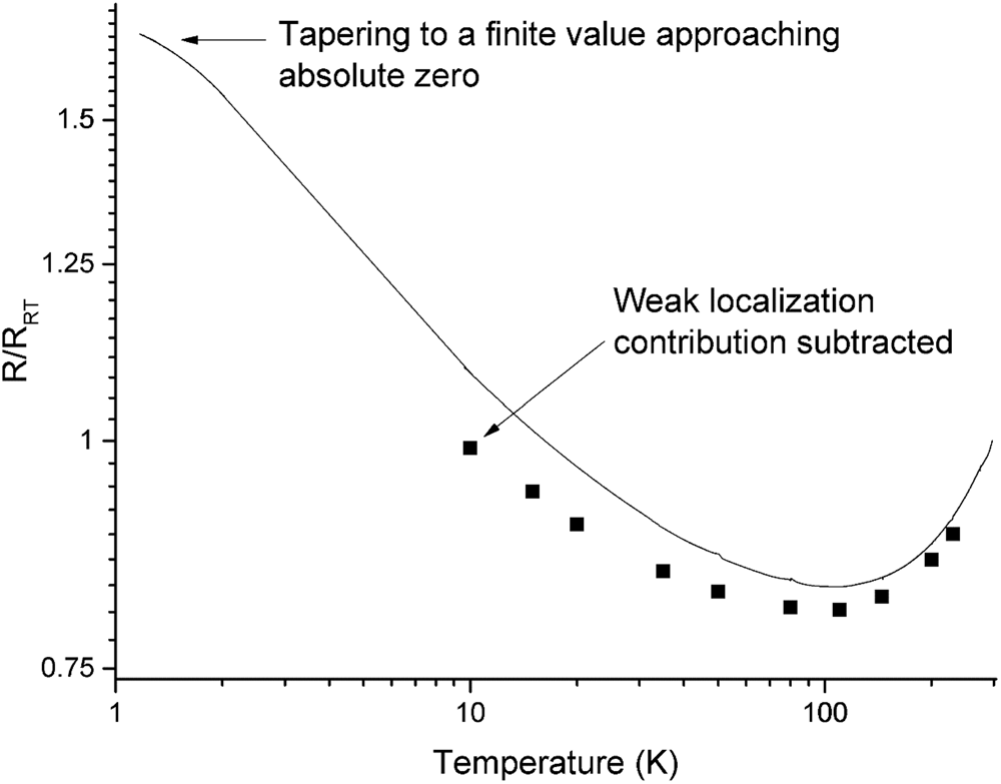
Log log plot of *R*/*T* for the aligned CNT fiber, represented by the solid black line and normalized by the room temperature resistance *R*_*RT*_. Squares indicate *R*/*T* values once weak localization effects are subtracted.

Now we consider the transport of as-is, aligned CNT fiber manufactured in house in a one-step floating catalyst chemical vapor deposition process. As discussed, there is a greater amount of residual catalyst and amorphous carbon in this material and the metallic SWCNT concentration is not controlled. The fiber however has high internal alignment and individually the CNTs are hundreds of times longer^26^ than in the unaligned SWCNT films. Figure 5 is the zero-field *R*/*T* plot. Above 110 K, *R* increases with increasing *T* like a metal. This manifests from the intrinsic CNT contribution over the fiber’s extrinsic factors, such as junctions and entanglement. Such metal-like behaviour has been observed from a variety of other CNT materials^44–46^ and conductive polymers^47,48^. The fiber’s metallic-like response is distinct from the unaligned SWCNT films, where we only observed a semi-conducting like temperature response. Below 110 K however, a crossover occurs where *R* now increases with decreasing *T* like a semi-conductor. Below 2 K, *R* begins to level-off to an apparently finite value approaching absolute zero. The level-off of *R* approaching absolute zero indicates the fiber sits on the metal side of the insulator to metal transition^6^ and contrasts the diverging resistance of the unaligned SWCNT films. This suggests that CNT length and alignment are more critical to obtaining metal-like conductivity over other production factors such as chemical treatment and metallic SWCNT concentration. This is likely because of the degree to which there is substantially fewer extrinsic junctions in a network of aligned, long length CNTs compared to an unaligned network of CNTs that individually are hundreds of times shorter. With the upcoming discussion of magneto-transport, we a priori eliminate magneto-transport models invoking hopping mechanisms because hopping is on the insulator side of the insulator to metal transition where charge carriers are localized.

### Aligned CNT Fiber High-Field Transport

**Figure 6:**
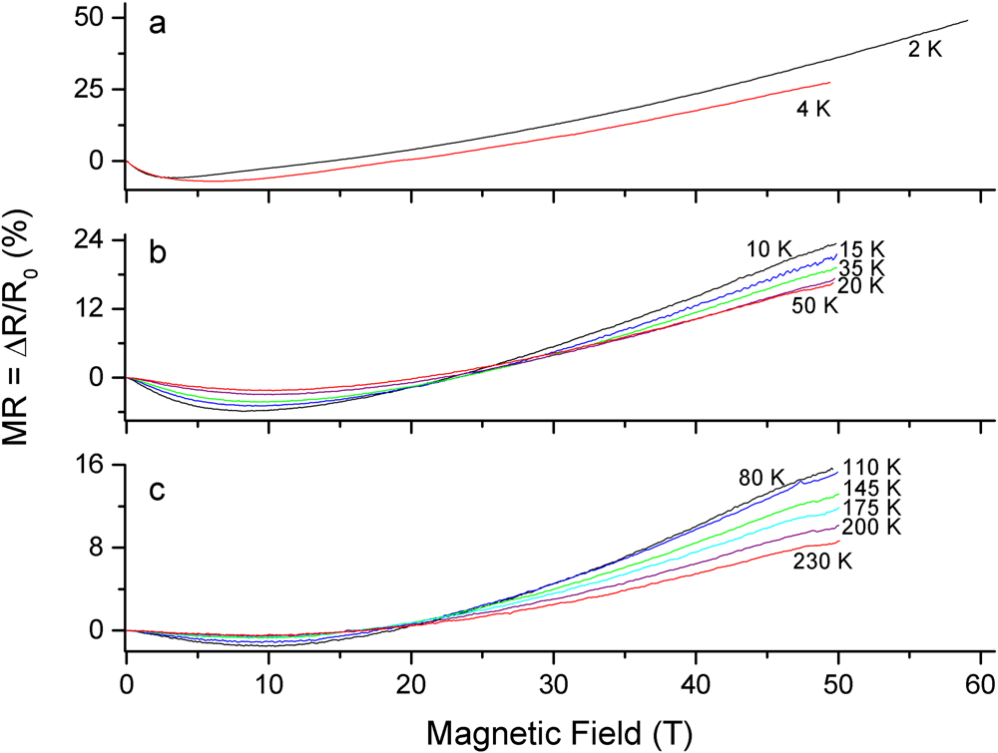
For clarity, the MR of the aligned CNT fiber is shown in three different temperature ranges: (**a**) 2–4 K, (**b**) 10–50 K, (**c**) 80–230 K. Note that the MR scales change on each plot. *R*_0_ is the resistance at zero-field. Note that this response contrasts the level-off behavior of the unaligned SWCNT films.

Figure 6 shows the magneto-resistance of the aligned CNT fiber, from 230 K to 2 K. As with the unaligned SWCNT films, MR is first negative and then turns positive at higher field. The positive MR component becomes greater at lower temperatures and is roughly quadratic with field. It is noteworthy that the MR does not saturate like the unaligned SWCNT films and indicates different transport mechanisms are present between these materials.

### High-Field Analysis

Now we analyse the high-field, positive MR component of the aligned CNT fiber. On the metal side of the insulator to metal transition where hopping mechanisms are excluded, there are two prevalent mechanisms leading to a positive MR reported in CNT literature: (1) thermally induced electron diffusion reduced by electron-electron interaction (Sometimes abbreviated EEI) and (2) the classical two band model. Thermally induced electron diffusion is regarded as a liquid helium temperature phenomenon^48,49^ and we see positive MR persisting to 230 K, the highest temperature we measured. While thermally induced electron diffusion cannot be ruled out for liquid helium temperatures, we require another mechanism to explain the positive MR at higher temperatures – the classical two band model. The two band model is a classical effect that results from the interaction of the magnetic field on moving charges. While widely applied to crystalline graphite and carbon fibers^13,50,51^, the inclusion of classical MR into CNT transport studies is infrequent and seems limited to the earliest CNT transport studies^52,53^. This is possibly from the unfavourable combination of low mobility in CNT materials and in-availability of sufficiently high-field. Another low-field magneto-transport study on similar aligned CNT materials^23^ suggested that positive MR was an electromechanical effect between the fiber, field, and residual catalyst, without discussing classical two band MR as a possibility. If catalyst interaction significant, we would expect to observe signs of magnetization saturation at high-field and this was not observed. Further, electromechanical interaction of the catalyst would not yield the realistic mobility data we will later show. Looking at Fig. 6, if we restrict consideration to fields found in typical cryogenic laboratories (~10 T), indeed the positive MR component only becomes obvious at helium temperatures and it is plausible to assign positive MR to other mechanisms; this highlights the necessity of the high-field study.

The general behaviour of this classical MR mechanism is another quadratic dependence, MR = *μ* ^2^ *H* ^2^ where *μ* is the charge carrier mobility. Particularly suited for single crystal graphite, this simple model assumes there is an equal density of holes *n*_*H*_ and electrons *n*_*E*_, and that their mobilities are equal. Further, (*µH*)^2^ ≪ 1^51^. In cases where electron and hole density are not equal (*n*_*E*_ ≠ *n*_*H*_) and when the field is not orthogonal to the graphene planes, the generalized expression is

5 $$MR=\frac{{\mu }^{2}{H}^{2}{\rm{\Theta }}}{1+{\mu }^{2}{n}^{2}{H}^{2}{\rm{\Theta }}}(1-{n}^{2})$$

where *n* is the ratio difference between charge carriers *n* = (*n*_*E*_ − *n*_*H*_)/(*n*_*E*_ + *n*_*H*_) and *Θ* ≈ Cos^2^ *θ* where *θ* is the angle between the graphene plane and field^51,52^. In single crystal graphite, it is possible to orient the field against all of the graphene planes. For more complicated morphologies, such as carbon fiber, this is not possible and consideration of *Θ* becomes necessary. Because of the tubular nature of the CNTs, the measured MR of the bulk CNT textile is the angular average of equation (5)^52^. This yields:

6 $$MR=(1-{n}^{-2})(\sqrt{\frac{1}{1+{H}^{2}{n}^{2}{\mu }^{2}}}-1)$$

Equation (6) was fitted to the high-field portion of the MR data, from approximately the start of the MR upturn to the highest fields measured across all temperatures. A constant parameter was added to account for the separate negative MR contribution that is present at low-field, say possibly weak localization, that is assumed to saturate to a constant value at high-field where the fitting of equation (6) starts.

**Figure 7:**
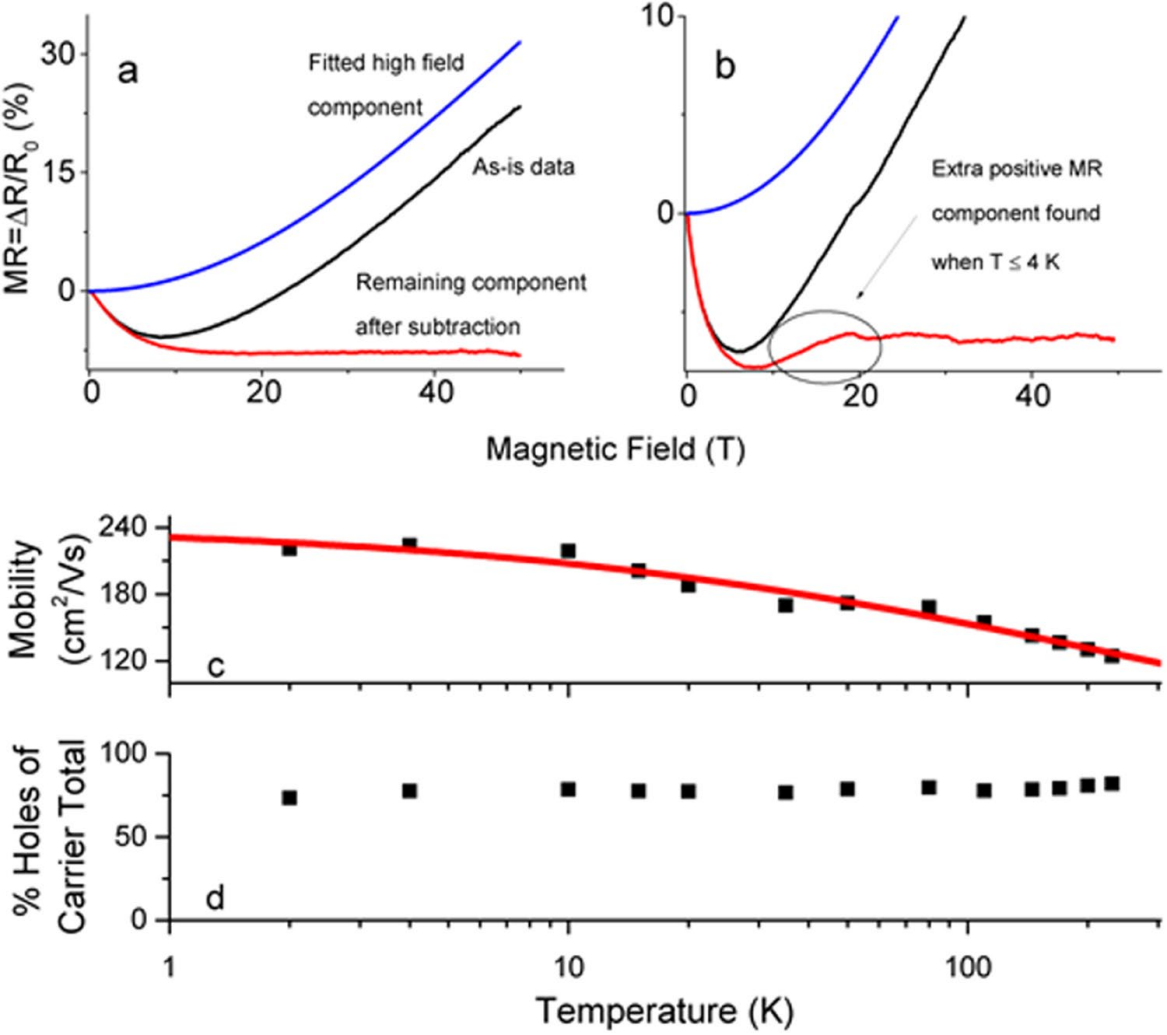
The simple two band model fits to the high-field portion of the MR data yielding transport data. (**a**) Example of high-field fitting at 10 K, which is representative for cases 10 K to 230 K. Subtracting the high-field fit from the measured data leaves a remaining negative MR contribution. (**b**) Example of fitting for 4 K, which is representative of fittings 4 K and below. This result is like the higher temperatures except there is a small positive MR component remaining after the fit is subtracted from the MR data, as indicated by the circle. (**c**) Carrier mobility as a function of temperature. The red line fit is from equation (7) ^51,52^ consisting of a temperature independent term (elastic collisions with crystal defects) and a temperature dependent term (inelastic collisions with either phonons or other charge carriers). (**d**) Temperature dependence of the charge carrier fraction showing that approximately 80% of the charge carriers are holes independent of temperature.

Figure 7 a shows a typical fit for the CNT fiber’s positive MR component. Also shown, this fit was subtracted from the measured data and this yields the negative MR component by itself. The fitted high-field component matches the high-field data and the remaining negative component indeed shows the expected saturation. This particular fitting is representative for temperatures spanning 10–230 K. Figure 7b shows an example of high-field fitting for temperatures 4 K and below. We found the high-field data still fits well to the simple two band model although the remaining component after subtraction still has a non-constant positive MR component remaining. This hints at thermally induced electron diffusion becoming prevalent at liquid helium temperatures and will be discussed later.

The classical two band model provides two fitting parameters, the charge carrier mobility *µ* and *n* the ratio of one carrier type out of the total. Figure 7c shows mobility *µ* as a function of temperature *T*. At the lowest temperatures, mobility is roughly temperature independent and then begins to decrease with increasing temperature. Similar behaviour has been seen with carbon fibers^51^ and CNTs^52^ where the total mobility is modelled with two components: a temperature independent component *µ*_*DEFECT*_ representing defect-induced boundary scattering and a temperature dependent power law term, *µ*_*PHONON*_ *T* ^−*x*^, representing phonon collision where *x* is an exponent. These terms add in parallel according to Matthiessen’s rule:

7 $$\mu (T)={(\frac{1}{{\mu }_{DEFECT}}+\frac{1}{{\mu }_{PHONON}{T}^{-x}})}^{-1}$$

The red trace in Fig. 7c shows the unconstrained best fit of equation (7) with *µ*_*DEFECT*_ = 240 +/− 14 cm^2^/Vs, *µ*_*PHONON*_ = 4920 +/− 2940 cm^2^/Vs, and *x* = 0.54 +/− 0.11, with an R-squared of 0.97 and an adjusted R-squared of 0.96. We stress that we are fitting a three-parameter model that is well-established in the transport of carbon fiber. Adding extra terms or permitting *µ*_*DEFECT*_ to have temperature dependence does not lead to a better fit. The exponent of the fitted temperature dependent term (x ~ 0.5) contrasts the value for typical phonon scattering (*x* = 1) in many materials, although is similar to values for optical phonon interaction^54^. In carbon literature, the most crystalline graphite forms have *x* ranging from 1 to 1.6^51^. Less crystalline graphitized carbon fiber however has been found to have similar temperate dependence to our case, with *x* ranging from 0.51 to 0.69^51^. They attributed *x* < 1 from an additional influence of defect-induced boundary scattering that is present even at room temperature.

The mobility data shows defect-induced boundary scattering is significant in our CNT fiber. The following equation estimates the average distance between elastic scattering sites: l_ELASTIC_ = *µ*(0) *v*_*F*_ *m*_*E*_ e^−1^ ^51^. Taking the mobility at absolute zero, *µ*(0) = *µ*_*DEFECT*_; the expected Fermi velocity, *v*_*F*_ = 8 * 10^5^ m/s^55^; the effective CNT electron mass, *m*_*E*_ = 7.8 * 10^−32^ kg^5^; and the electric charge *e*, we estimate the distance between elastic scatting sites *l*_*ELASTIC*_ ~ 10 nm. This scattering distance is far smaller than the distance between CNT junctions (assumed to be the CNT length, ~1 mm) and shows that crystal defects on the CNTs themselves limit the mobility and overall transport, not just the extrinsic CNT junctions. Note that *l*_*ELASTIC*_ ~ 10 nm compares to the mean free path length found in other high performance bulk CNT fibers, measured by different means to be approximately 32 nm to 38 nm^56^.

The fraction of one charge carrier over the total, *n*, is the other fitted parameter from the classical two band model (Fig. 7d). The CNT fiber is not chemically treated except for atmospheric exposure, which is well known to *p* dope CNT materials^57^. We observe in Fig. 7d that across the entire temperature range studied, 80% of the conduction comes from holes and 20% from electrons. Addressing how this 80/20 ratio could be the case, presume that the various CNT electronic species contribute to MR as an un-weighted average. A metallic CNT will have half electrons and half holes. A fully doped semi-conducting CNT will be primarily hole conduction because chemical *p* doping shifts the Fermi level into the Valence band. Although often a too simple picture for practical chemical vapor deposition, assume that over the distribution of CNT diameters generated, chirality is randomly distributed and 1/3 of the CNTs are metallic and 2/3 are semi-conducting. The expectation value for the hole fraction is (1/3)(50%) + (2/3)(100%) = 83%. This is very close to the fitted *n* value and, if this assumption is valid, stronger doping should edge the fitted value closer to, but not over this limit. A fiber consisting of purely metallic CNTs should have a fraction of 50% independent to doping degree.

### Low-Field Analysis

Subtraction of the positive high-field MR component from the data set enabled an unobscured analysis of just the low-field negative MR component. Temperature and field dependent analysis shown in the supplemental section (Figs S5–1 and S5–2) supported that this negative MR component originated from two dimensional weak localization, a quantum mechanical effect widely used to explain negative MR in disordered thin film metals^17,18^, disordered graphite^19,20,58^, and CNTs^2,6,49,52,59–61^. Weak localization’s two dimensional nature manifests from charge carrier restriction on the surface of a CNT bundle, which requires that weak localization’s characteristic length, the coherence length *L*_φ_, is greater than the CNT bundle diameter. This is shown in supplemental section (Figs S5–2) where, with increasing temperature, the measured coherence length *L*_φ_ decreases and the related dephasing field, *H*_φ_, increases according to a power law. With increasing temperature *L*_φ_ reaches 30 nm, the approximate width of the CNT bundles (Fig. 1), and a departure from the power law is observed. While more high temperature data is required, this is possibly a temperature dependent cross-over from two dimensional to three dimensional weak localization and has been observed before in CNT transport^61^.

Weak localization contributes a relatively small, zero-field resistance increase as temperature is lowered. The squares in the zero-field *R*/*T* plot (Fig. 5) shows the two dimensional weak localization contribution subtracted from the resistance. While weak localization explains the low-field negative MR, as shown its contribution is small to the overall zero-field fiber resistance–primarily consisting of an extrinsic contribution (from voids, impurities, and CNT junctions) and an intrinsic contribution (from scattering on the CNTs themselves).

It is worthy to note that the fluctuation induced tunneling model describes the extrinsic resistance contribution of voids, impurities and junctions within many bulk CNT materials^31,62,63^ on the metal side of the insulator to metal transition. Typically, it accounts for most of the zero-field, room temperature resistance of these materials and, in the aligned CNT fiber considered here, fluctuation induced tunneling accounts for ~80% of the room temperature resistance (see supplemental information, Figs S6–1). The magnetic field dependence of fluctuation induced tunneling is an open question in the literature^31^, although there is some speculation that the field induced shrinking wave function from hopping conduction may qualitatively apply^30^. Here we point out that the MR response of the aligned CNT fiber above 10 K is accounted for with solely the classical two band model and weak localization; this supports that fluctuation induced tunneling itself has no magnetic field dependence. Fluctuation induced tunneling is a function of the distance between CNT structures and the tunneling potential between them, as dictated by surface chemistry^64^. To the degree these factors are not influenced by field, it is plausible fluctuation induced tunneling has no field dependence.

## Conclusion

High-field magneto-transport was measured for two types of CNT materials: (1) unaligned and purified SWCNT films of controlled metallic concentration and chemical treatment and (2) less pure, but internally aligned long length CNTs forming a bulk fiber. The high-field transport going up to 60 T was necessary to differentiate between high-field transport mechanisms. For studies restricted to fields found in typical cryogenic laboratories, the selected magneto-transport model would not necessarily be an obvious choice.

The temperature response of the metallic, semi-conducting and unsorted unaligned films all showed hopping behavior. Standard acid treatment increased the conductivity of all films and brought the unsorted and metallic films closer to the insulator to metal transition. The acid treated, unsorted SWCNT film was the most conductive, even more than the acid treated metallic SWCNT film. The acid induced conductivity increase of the metallic SWCNT film illustrates the influence of extrinsic junctions and how they may be mitigated.

The MR response of unaligned SWCNT films distinctly saturated at high-field, indicating that saturation from spin polarization was responsible for positive MR. The other prevalent positive MR mechanism, within the variable range hopping framework, was field induced wave function shrinking and this mechanism did not seem to apply here. Acid treatment suppressed the magnitude of the positive MR component. In terms of the spin saturation model, this suppression is explained with an increase of localization length and carrier density. The as-is unsorted film displayed a secondary, negative MR component in high-field that was not present after chemical treatment, or with the predominantly metallic or semi-conducting SWCNT films. This secondary negative MR will require further investigation.

The aligned, high performance CNT fiber was on the metal side of the insulator to metal transition with a distinct MR response that did not saturate. A classical two band model fits the high-field data well and yields realistic and relevant transport parameters. Appropriately fitting the high-field component enabled its subtraction from the measured data. This permitted unconfounded low-field analysis, which was shown to be two-dimensional weak localization dictated by CNT bundle interfaces. Further, we point out that while weak localization and the two band model accounts for the magneto-resistance, most of the fiber’s zero-field resistance comes from extrinsic contributions as modeled by fluctuation induced tunneling. This implies that fluctuation induced tunneling has no magneto-resistance, which this was an open question in the literature. Ultimately, we found that high magnetic field is an excellent probe for bulk mobility and crystallinity. As crystallinity of carbon nanotubes improves, it is possible other traditional techniques such as Raman spectroscopy and X-ray diffraction could lose resolution as was the case for graphitic intercalation compounds^13^. High-field transport may become a necessary characterization tool for highly crystalline CNT based textiles.

### Availability of Datasets

The datasets analyzed in this study are predominately included in this published article and the Supplementary Information. Otherwise it is available from the corresponding author on reasonable request.

## Electronic supplementary material

Supplemental Information

## References

[1] Lekawa-Raus A, Patmore J, Kurzepa L, Bulmer J, Koziol K (2014). Electrical properties of carbon nanotube based fibers and their future use in electrical wiring. Adv. Funct. Mater..

[2] Yanagi K (2010). Transparent mechanisms in metallic and semiconducting single-wall carbon nanotube networks. ACS Nano.

[3] Blackburn JL (2008). Transparent conductive single-walled carbon nanotube networks with precisely tunable ratios of semiconducting and metallic nanotubes. ACS Nano.

[4] Jackson RK, Munro A, Nebesny K, Armstrong N, Graham S (2010). Evaluation of transparent carbon nanotube networks of homogeneous electronic type. ACS Nano.

[5] Salvato M (2011). Effect of potassium doping on electrical properties of carbon nanotube fibers. Phys. Rev. B.

[6] Vavro J, Kikkawa JM, Fischer JE (2005). Metal-insulator transistion in doped single-wall carbon nanotubes. Phys. Rev. B.

[7] Zhao Y, Wei J, Vajtai R, Ajayan P, Barrera E (2011). Iodine doped carbon nanotube cables exceeding specific electrical conductivity of metals. Sci. Rep..

[8] Behabtu N (2013). Strong, Light, Multifunctional Fibers of Carbon Nanotubes with Ultrahigh Conductivity. Science.

[9] Wang J, Luo X, Wu T, Chen Y (2014). High-strength carbon nanotube fibre-like ribbon with high ductility and high electrical conductivity. Nat. Commun..

[10] Bucossi A (2015). Enhanced Electrical Conductivity in Extruded Single-Wall Carbon Nanotube Wires from Modified Coagulation Parameters and Mechanical Processing. ACS Appl. Mater. Interfaces 2015.

[11] Shioya J, Matsubara H, Murakami S (1986). Properties OF AsFs Intercalated Vapor Grown Graphite. Synth. Met..

[12] Inagaki M (1989). Applications of graphite intercalation compounds. J. Mater. Res..

[13] Chieu TC, Timp G, Dresselhaus MS, Endo M, Moore AW (1983). High-field magnetoresistance measurements on highly ordered graphite fibers. Phys. Rev. B..

[14] Bulmer J, Gspann T, Barnard J, Elliott J (2017). Chirality-independent characteristic crystal length in carbon nanotube textiles measured by Raman spectroscopy. Carbon.

[15] Jaiswal M, Wang W, Fernando K, Sun Y, Menon R (2007). Charge transport in transparent single-wall carbon nanotube networks. J. Phys.: Condens. Mat..

[16] Jaiswal M, Wang W, Fernando K, Sun Y, Menon R (2007). Magnetotransport in transparent single-wall carbon nanotube networks. Phys. Rev. B..

[17] Bergmann G (1984). Weak localization in thin films - a time-of-flight experiment with conduction electrons. Phys. Rep..

[18] Lee PA, Ramakrishnan T (1985). Disordered electronic systems. Rev. Mod. Phys..

[19] Bayot V (1990). Two-dimensional weak localization in partially graphitic carbons. Phys. Rev. B..

[20] Bayot V, Piraux L, Michenaud J-P, Issi J-P (1989). Weak localization in pregraphitic carbon fibers. Phys. Rev. B..

[21] Arnold MS, Green AA, Hulvat JF, Stupp SI, Hersam MC (2006). Sorting carbon nanotubes by electronic structure using density differentiation. Nat. Nanotechnol..

[22] “NanoIntegris”. [Online]. Available: http://www.nanointegris.com/. [Accessed 29 03 (2017)].

[23] Cimpoiasu E, Sandu V, Levin G, Simpson A, Lashmore D (2012). Angular magneto-resistance of streched carbon nanotube sheets. J. Appl. Phys..

[24] Nirmalraj PN, Lyons PE, De S, Coleman JN, Boland JJ (2009). Electrical connectivity in single-walled carbon nanotube networks. Nano Lett..

[25] Ahlskog M, Reghu M, Heeger A, Noguchi T, Ohnishi T (1996). Noguchi and T. Ohnishi Electronic transport in the metallic state of oriented poly p-phenylenevinylene. Phys. Rev. B..

[26] Koziol K (2007). High-performance carbon nanotube fiber. Science.

[27] Shiraishi M, Ata M (2003). Tomonaga–Luttinger-liquid behavior in single-walled carbon nanotube networks. Solid State Communications.

[28] Barnes TM, Blackburn JL, Lagemaat Jvd, Coutts TJ, Heben MJ (2008). Reversibility, dopant desorption, and tunneling in the temperature-dependent conductivity of type-separated, conductive carbon nanotube networks. ACS Nano.

[29] Takano T, Takenobu T, Iwasa Y (2008). Enhancement of carrier hopping by doping single walled carbon nanotube films. J. Phys. Soc. Jpn.

[30] Salvato M (2011). Low temperature conductivity of carbon nanotube aggregates. J. Phys.: Condens. Matter.

[31] Ksenevich V, Odzaev V, Martunas Z, Seliuta D, Valusis G (2008). Localization and nonlinear transport in single walled carbon nanotube fibers. J. Appl. Phys..

[32] Fuhrer M (1999). Nonlinear transport and localization in single-walled carbon naotubes. Synthetic Met..

[33] Rosenbaum R, Murphy T, Palm E, Hannahs S, Brandt B (2001). Magnetoresistance of insulating amorphous NixSi(1−x) films exhibiting Mott variable-range hopping laws. Phys. Rev. B.

[34] Kurobe A, Kamimura H (1982). Correlation effects on variable range hopping conduction and the magnetoresistance. J. Phys. Soc. Jpn..

[35] Frydman A, Ovadyahu Z (1995). Spin and quantum interference in hopping conductivity. Solid State Commun..

[36] Len T (2011). Magnetoresistance of nanocarbon materials. Low Temp. Phys..

[37] Demishev SV (2008). Scaling of magnetoresistance of carbon nanomaterials in Mott-type hopping conductivity region. Phys. Solid State.

[38] Fedotov A, Prischepa S, Danilyuk A, Svito I, Zukowski P (2014). Spin-polarized and normal hopping magnetoresistance in heavily doped silicon. Acta Phys. Pol. A.

[39] Yosida Y, Oguro I (1999). Variable range hopping conduction in bulk samples composed of single-walled carbon nanotubes. J. Appl. Phys..

[40] Yosida Y, Oguro I (1998). Variable range hopping conduction in multiwalled carbon nanotubes. J. Appl. Phys..

[41] Nguen V, Spivak B, Shklovskii B (1985). Tunnel hopping in disordered systems. Sov. Phys. JETP.

[42] Schirmacher W (1990). Quantum-interference magnetoconductivity in the variable-range-hopping regime. Phys. Rev. B.

[43] Sivan U, Entin-Wohlman O, Imry Y (1988). Orbital magnetoconductance in the variable-range-hopping regime. Phys. Rev. Lett..

[44] Ahn S, Nam YKY, Yoo H, Park J, Park Y (2009). Magnetotransport in iodine-doped single-walled carbon nanotubes. Phys. Rev. B.

[45] Kaiser A, Dusberg G, Roth S (1998). Heterogeneous model for conduction in carbon nanotubes. Phys. Rev. B.

[46] Skakalova V, Kaiser A, Woo YS, Roth S (2006). Electronic transport in carbon nanotubes: from individual nanotubes to thin and thick networks,”. Phys. Rev. B.

[47] Kaiser A (1991). Metallic behavior in highly conducting polymers. Synthetic Met..

[48] Kaiser A (2001). Electronic transport properties of conducting polymers and carbon nanotubes. Rep. Prog. Phys..

[49] Choudhury PK, Jaiswal M, Menon R (2007). Magnetoconductance in single-wall carbon nanotubes: Electron-electron interaction and weak localization contributions. Phys. Rev. B.

[50] Soule DE (1958). Magnetic field dependence of the hall effect and magnetoresistance in graphite single crystals. Phys. Rev..

[51] Woolf LD, Chin. J, Lin-I.iu. YR, Ikezi H (1984). Electrical transport properties of benzene-derived graphite fibers. Phys. Rev. B.

[52] Song SN, Wang XK, Chang RPH, Ketterson JB (1994). Electronic properties of graphite nanotubules from galvanomagnetic effects. Phys. Rev. Lett..

[53] Baxendale M, Mordkovich VZ, Yoshimura S, Chang RPH (1997). Magnetotransport in bundles of intercalated carbon nanotubes. Phys. Rev. B.

[54] Yao Z, Kane CL, Dekker C (2000). High-field electrical transport in single-wall carbon nanotubes. Phys. Rev. Lett..

[55] Beard M, Blackburn J, Heben M (2008). Photogenerated free carrier dynamics in metal and semiconductor single walled carbon nanotube films. Nano Lett..

[56] Tristant D (2016). Enlightening the ultrahigh electrical conductivities of doped double-wall carbon nanotube fibers by Raman spectroscopy and first-principles calculations. Nanoscale.

[57] Collins P, Bradley K, Ishigami M, Zettl A (2000). Extreme oxygen sensitivity of electronic properties of carbon nanotubes. Science.

[58] Piraux L (1990). Weak localization and coulomb interaction in graphite intercalation compounds and related materials. J. Mater. Res.

[59] Cai J (2006). Pressure-induced transition in magnetoresistance of Single-walled carbon nanotubes. Phys. Rev. Lett..

[60] McIntosh G (2002). Orientation dependence of magneto-resistance behaviour in a carbon nanotube rope. Thin Solid Films.

[61] Salvato M (2012). Weak localization and dimensional crossover in carbon nanotube systems. Eur. Phys. J. B.

[62] Bhatia R, Prasad V, Menon R (2011). Probing the inter-tube transport in aligned and random multiwall carbon nanotubes. J. Appl. Phys..

[63] Kaiser A, Skakalova V, Roth S (2007). Modelling conduction in carbon nanotube networks with different thickness, chemical treatment and irradiation. Physica E.

[64] Sheng P (1980). Fluctuation-induced tunneling conduction in disordered materials. Phys. Rev. B.

