# Supplementary material for: Extreme Magneto-transport of Bulk Carbon Nanotubes in Sorted Electronic Concentrations and Aligned High Performance Fiber
Source: Sci Rep. 2017 Sep 22;7:12193. doi: 10.1038/s41598-017-12546-6 (PMC5610196; doi:10.1038/s41598-017-12546-6)
Supplement: Supplementary file 1 — Supplemental Information [file 41598_2017_12546_MOESM1_ESM.pdf]

Supplemental Information for

Extreme Magneto-transport of Bulk Carbon Nanotubes in Sorted Electronic Concentrations and Aligned High Performance Fiber

John S. Bulmer, Agnieszka Lekawa-Raus, Dwight G. Rickel, Fedor F. Balakirev, Krzysztof K. Koziol

S1-- Fitting Hopping Conduction. Qualitatively for the as-is unaligned SWCNT films, the asymptotic increase of resistance *R* as temperature *T* approaches absolute zero is a strong indicator of the variable range hopping (VRH) model. Following are the model fits to variable range hopping for the as-is unaligned SWCNT films. Although they have not been deliberately chemically treated, they have doping from atmosphere exposure. Here is the temperature dependent variable range hopping equation

$$R(T) = R_{0T}\exp\left[\left(\frac{T_{MOTT}}{T}\right)^{q}\right] \tag{S1}$$

where R_{0T} is the high temperature limit of the resistance, and q equals ½ for ES variable range hopping, 1/3 for two dimensional variable range hopping, and ¼ for three dimensional variable range hopping. The fits show that the as-is 98% semi-conducting film fits best to ES- variable range hopping, the unsorted film fits best to two dimensional variable range hopping, and the 95% metallic film fits bests to three dimensional variable range hopping. These results are largely consistent with literature [1] [2].

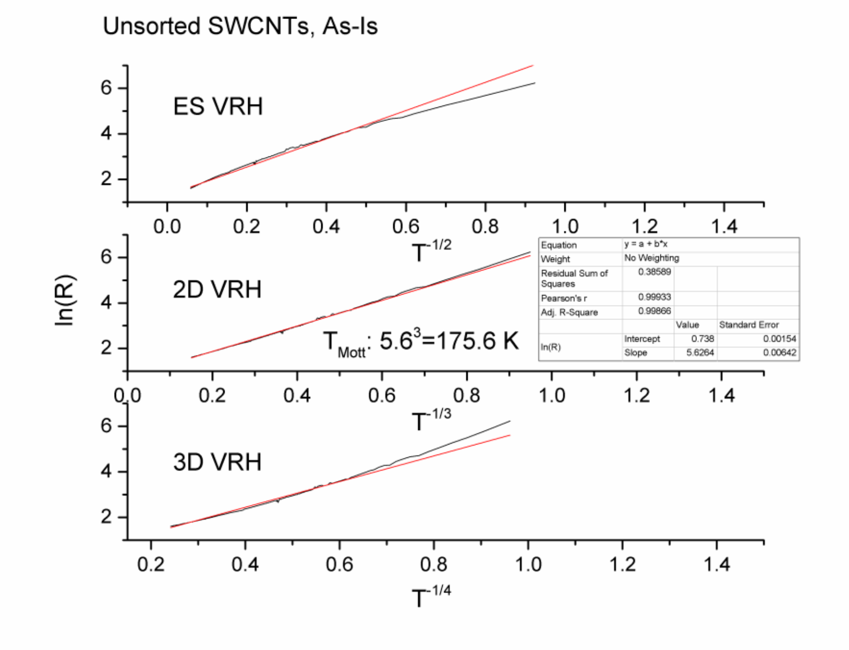

Figure S1-1| Two dimensional variable range hopping gives the best fit for the as-is unsorted unaligned SWCNT film. Black is the data and red is the fit.

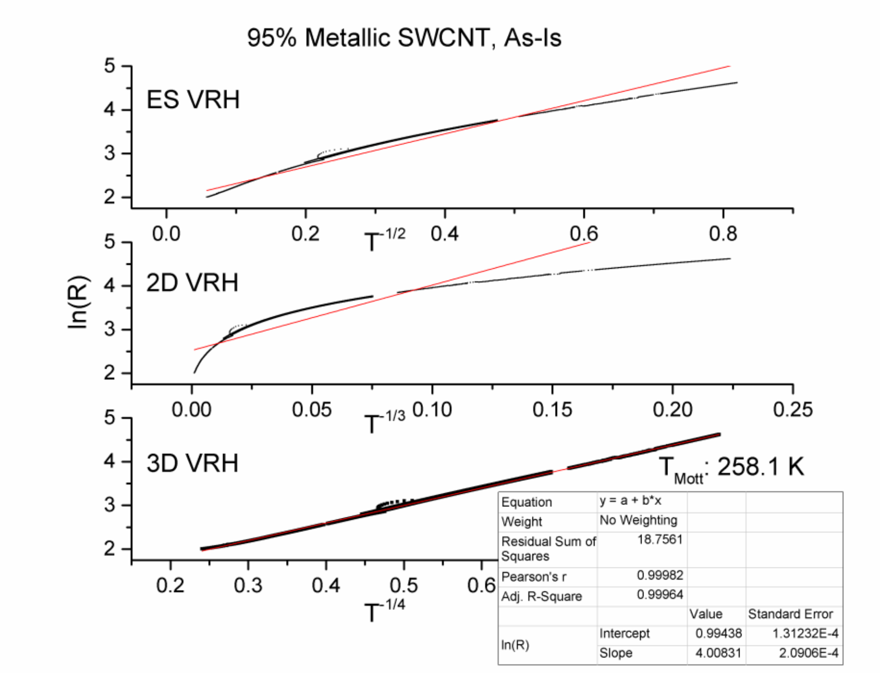

Figure S1-2| Three dimensional variable range hopping gives the best fit for the as-is metallic unaligned SWCNT film. Black is the data and red is the fit.

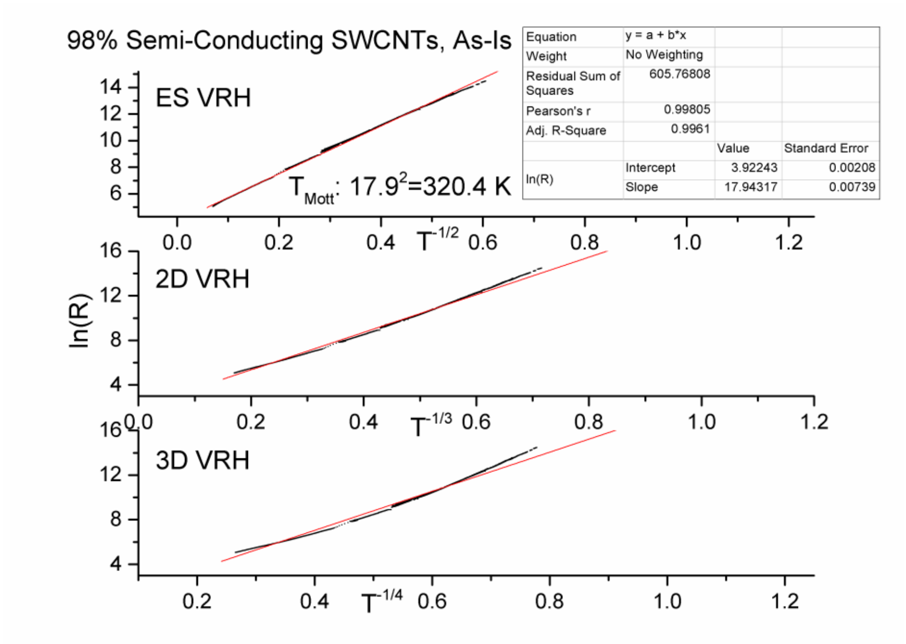

Figure S1-3| ES variable range hopping gives the best fit for the as-is 98% semi-conducting unaligned SWCNT film. Black is the data and red is the fit.

S2- Discrepancy with the MR saturation value. Figure S2-1 plots MR_{SAT} against temperature T in a way that linearizes equation (3) in the main text, the MR saturation equation. The fitted exponents are -0.39 (for the metallic SWCNT film) and -0.82 (for the unsorted SWCNT film), whereas the spin saturation model expects exponents of -1/4 [3] and -1/3 [4] respectively. As explained in the main text, this disagreement is from the offset of another negative MR mechanisms.

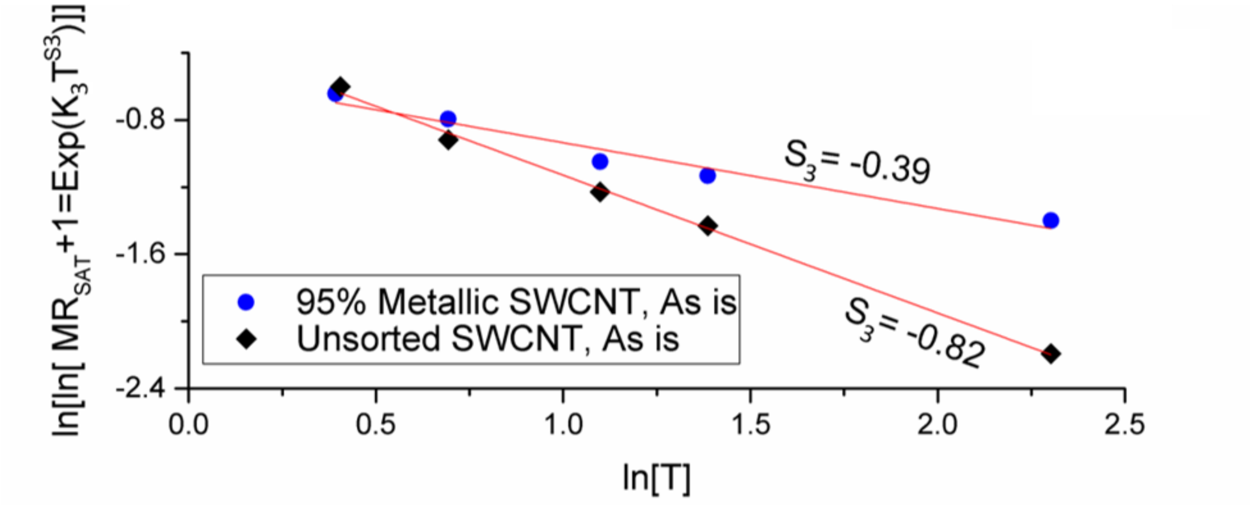

Figure S2-1| The saturated MR value, MR_{SAT}, as a function of temperature T, displayed in a way that linearizes equation (3) from the main text. Note that a separate, negative MR contribution makes the measured value lower than that predicted by the spin saturation model.

S3- Fitting the shrinking wave function model for the unaligned SWCNT films. As previously discussed for the unaligned SWCNT films, the low-field data comprises of several transport mechanisms that individually have negative MR and positive MR contributions. This complicates analysis of the low-field data, although it is expected the negative MR component saturates to a steady-state, field-independent value. For the field induced shrinking wave function model, the natural log of MR is linear with H^2 in the low-field limit (see equation 1 in the main text) and, when plotting against H^2, such a relatively linear region appears consistently soon after the MR swings from a negative direction to the positive one. This linear region is taken as the low-field limit of the proposed shrinking wave function model where it is assumed the negative MR contribution has saturated. An example is shown in the inset of figure S3-1. The slope of this linear portion is plotted against T in the log-log plot below to determine the applicability of the shrinking wave function model. Fitted exponents of the power law were 2.97 (for the metallic unaligned SWCNT film) and 2.04 (for the unsorted unaligned SWCNT film), where the shrinking wave function model expects 3/4 (for three dimensional hopping [5]) and 1 (for two dimensional hopping [6]). The quality of these linear fits are not as good and the fitted parameters are far off the theoretical values, at least compared to the fit quality of the rivalling spin saturation model. Thus, the fit of the spin saturation model from higher field data is better than the fit of the wave function shrinking model from lower field data.

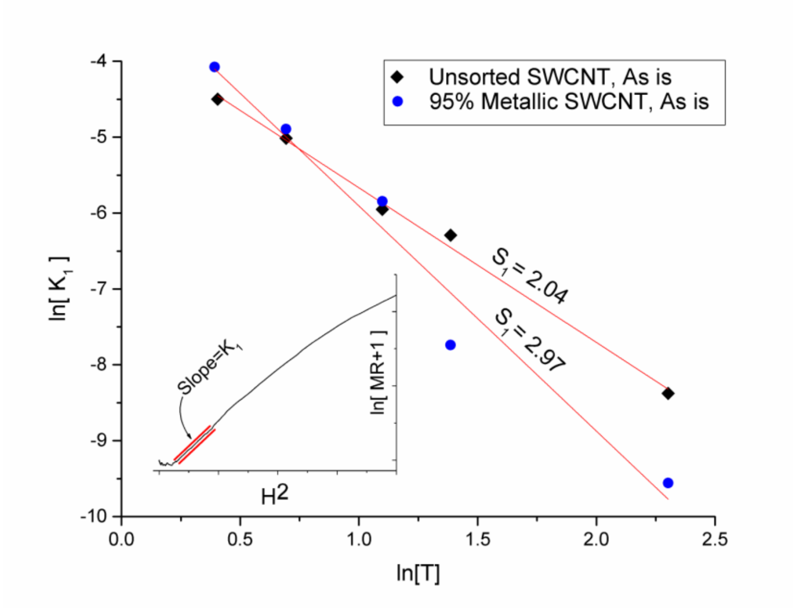

Figure S3-1| Inset, to demonstrate applicability of the wave function shrinking model, a typical example of MR data is plotted against H^2 and the slope of the linear region is extracted. **Main**, this slope, collected for a range of temperatures, is plotted against temperature in a log-log plot.

S4—Fitting the field saturation value, H_{SAT}, for the insulator to metal transition. As discussed in the text, chemical treatment brought the metallic and unsorted SWCNT films close to the insulator to metal transition. The spin saturation model is for variable range hopping and it seems that the theory has not been extended to the insulator to metal transition yet. Still, we plot H_{SAT} against T a way to reveal power laws.

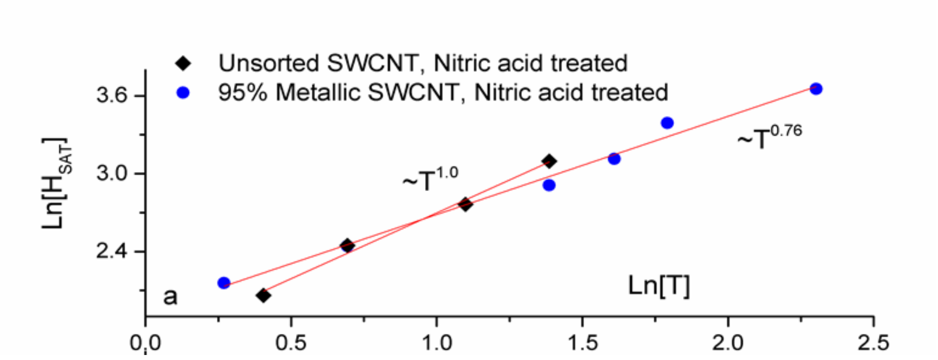

Figure S4-1| a, the field saturation values, H_{SAT}, plotted against temperature T on a log-log plot for the unaligned SWCNT films when chemically treated.

S5- Weak Localization. We next discuss the low-field, negative MR that remains after subtraction of the high-field MR component. We stress that correct, unconfounded analysis of the negative MR at low-field would not have been possible if the high-field data had not been collected and subtracted out. For the purposes of discussion, assume from now on that this positive MR component has been subtracted. Figure S5-1 shows the saturated value of the negative MR component, converted to the absolute change of conductance $\Delta G=R(H)^{-1}-R(0)^{-1}$, as a function of logarithmic temperature. From 10 to 230 K, $\Delta G \propto \mathrm{Ln}[T]$, which is consistent with two dimensional weak localization [1] [7] [8]. Fitting to three dimensional weak localization temperature dependent models [2] [9] had appreciably lower success. For $T \leq 4$, the Ln[T] dependence is lost and indicates an additional mechanism limiting transport at liquid helium temperatures. This is consistent with thermally induced electron diffusion reduced by electron electron interaction (EEI), particularly because there is also a positive MR component distinctly remaining after the primary positive MR component is subtracted (in main text, for $T \leq 4$, see figure 7b).

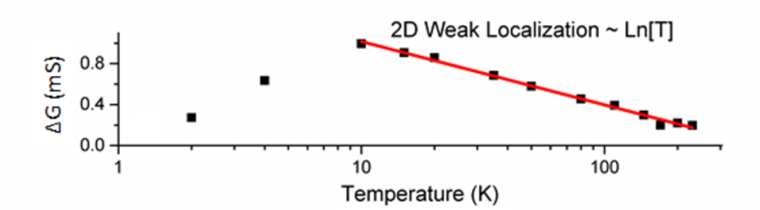

Figure S5-1| The saturated values of the subtracted negative MR component, in terms of ΔG. The linear fit on the log plot is an indicator that two dimensional weak localization is responsible for the negative MR response, at least from 10- 230 K.

The squares in figure 5 from the main text shows this two dimensional weak localization contribution subtracted from the R/T plot. While weak localization explains the negative MR, as shown its contribution is small to the overall fiber resistance at zero field-- primarily consisting of a large extrinsic contribution (from voids, impurities, and CNT junctions) and an intrinsic contribution (from scattering on the CNTs themselves).

Weak localization is a quantum mechanical phenomenon that explains the small resistance increase in disordered metallic thin films as they are cooled to cryogenic temperatures [7] [10]. Later, the mechanism was used to explain the unusual electronic transport of turbostatic/ disordered graphite and carbon fiber [11] [12] [13] and then CNTs [1] [2] [8] [14] [15] [16] [17].

It comes about from the constructive interference of charge carrier wave functions backscattering elastically off crystal defects. Inelastic collisions with phonons tend to dephase the backscatter and, thus, increasing temperature suppresses weak localization's positive addition to resistivity. Applying a magnetic field also de-phases the backscatter, leading to a negative MR. L_φ is the coherence length, the average distance a charge carrier wave function looses coherence due to inelastic collisions. It is related to temperature and field via the following equation [8] [10] [17] [18]:

$$L_\phi = \sqrt{\frac{\hbar}{4eH_\phi}} = \sqrt{D_{iff}\tau} \propto T^{-p/2} \tag{S2}$$

where $\hbar$ is Plank's constant divided by 2π, e is the electric charge, H_φ is the dephasing field that destroys the backscatter coherence. D_{iff} is the diffusion constant and τ is the relaxation time between inelastic collisions. p is an exponent dependent on the exact inelastic scattering mechanism and the dimensionality of the transport. Because mobility μ is proportional to τ, it is expected that

$$\mu \propto T^{-p} \tag{S3}$$

In addition to its temperature dependence, the field dependence of the remaining negative MR component also supports a weak localization mechanism. Weak localization is a scaling theory meaning that all weak localization curves will match some universal curve f such that $\Delta\sigma = C f(H/H_\varphi)$, where C is a scaling parameter and the second scaling parameter is the dephasing field H_φ [2] [15]. Figure 5-S2 a shows the negative MR component curves at different temperatures aligning to a universal curve, here assigned as the one at 10 K. MR curves with temperatures less than 10 K are not considered here because of the expected confounding influence of EEI interaction. Using this scaling approach, the temperature dependent scaling parameter, H_φ, is plotted against temperature relative to H_φ at 10 K (Figure S5-2 c). The dephasing field H_φ follows the power law T^p where the power law exponent is p= 0.52 and then departs from this power law behavior at higher temperatures. From equation (S3), this means $\mu \propto T^{-0.52}$ and this matches the temperature dependent mobility component calculated independently from the high-field MR data.

Moving beyond the generalized scaling approach, for thoroughness, we fit the subtracted negative MR component to specific field dependent expressions for weak localization, given in [19] [20] for two dimensional weak localization and [10] [14] [21] for three dimensional weak localization. Figure S5-2b shows an example of a two dimensional weak localization fit to the low-field component at 10 K, which is representative across the temperatures measured 10 K and above. Figure S5-2d shows the fitted dephasing field parameter H_φ as a function of temperature, which is similar to the scaling approach except it is now in absolute terms and is not normalized. Note that results between the two different analytical approaches, fitting and scaling, nearly agree in terms of the power law dependence and the departure from the power law at higher temperatures. Fitting to three dimensional weak localization expressions across the temperature range was not as effective. It is noteworthy that weak localization, an effect based in quantum mechanics, is present approaching room temperature. While weak localization was originally applied to thin film metals for conductivity corrections below ~25 K [7], it has been shown to apply to pre-graphitic carbon materials approaching room temperature as well [12]. The presence of weak localization implies that elastic collisions between defects are more probable than phonon interactions and indicates the extent of crystal imperfection on the CNT structure.

Weak localization in two dimensions means that only two spatial coordinates are required to specify localization of the wave function. In CNT materials, two dimensional weak localization has been attributed to confinement of the charge carrier wave function on the surface of the CNT bundle [1] [17] , the natural aggregation of individual CNTs held together by van der Waals forces. In this picture, bundle bundle interfaces account for most of the overall dissipation; transport between CNTs within a bundle has little influence on the transport. Two dimensional weak localization applies provided the coherence length L_φ is larger than the bundle diameter, otherwise it is three dimensional [17]. Figure S5-2 e shows L_φ as a function of temperature, calculated from H_φ and equation (S2). At 10 K, L_φ = 60 nm and decreases with temperature until L_φ appears to level-off approaching 30 nm, which is a typical approximate value for bundle diameters in the fiber (Figure 1 from the main text). Because the trajectory of L_φ changes when the coherence length reaches the approximate bundle diameter, it is possible this is a cross-over from two dimensional to three dimensional weak localization (where a change in power law is expected). Similar cross-overs have been seen before in CNT systems [17], although in our case more analysis and model fitting on higher temperature data will be required to confirm the cross-over and a possibly

temperature dependent dimensionality. Conclusive determination of dimensionality at a given temperature could be made by changing the field direction relative to the current flow. Two dimensional weak localization is anisotropic and is strongest when the field is perpendicular to the current flow [7]; three dimensional weak localization is isotropic and field direction does not matter [10].

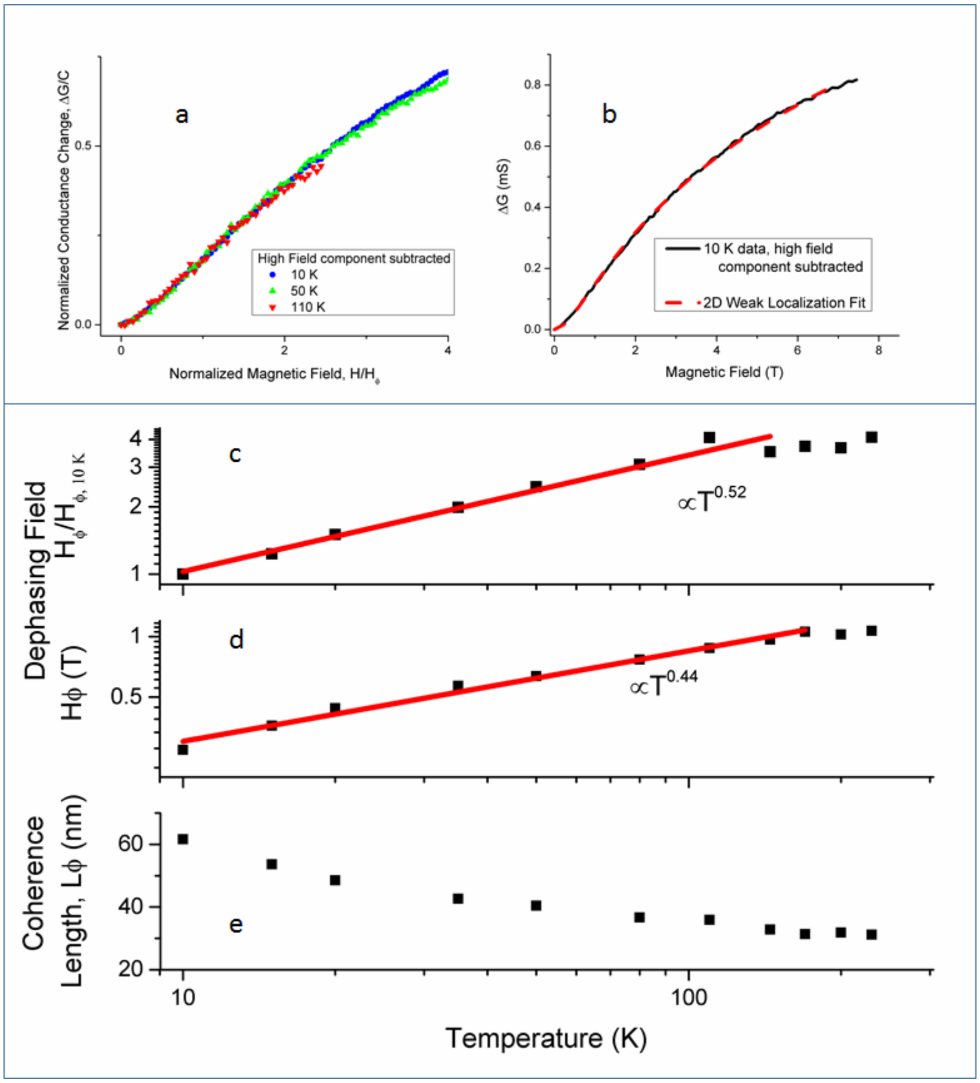

Figure S5-2| Weak localization fitting of the low-field MR data, after the positive MR component is subtracted. **a**, Examples at various temperatures of the scaling approach—the low-field MR component collapsing to a universal curve by adjusting the scaling parameters, *C* and H_φ. **b**, Example of a direct fitting of a low-field MR component to a specific expression of two dimensional weak localization. **c**, From the scaling approach, relative dephasing field as a function of temperature. **d**, From specific fitting of two dimensional weak localization, the dephasing field in absolute terms as a function of temperature. **e**, The coherence length, $L\varphi$, calculated from the dephasing field.

S6- Fluctuation Induced Tunnelling. This next section shows various fits for the fluctuation induced tunneling model on resistance *R* versus temperature *T* data of the aligned CNT fiber, zero field. Different fitting situations are considered, such as exclusion of temperatures equal to and less than 4 K. This was attempted because of concern about EEI interaction becoming significant and confounding the data. Another situation considered was the contribution from weak localization subtracted from the data. Two different metallic terms were considered, the quasi 1D metallic term and the standard metallic term. From these fits, the extrinsic contribution accounts for approximately 80% of the room temperature resistance. This was accomplished according to the equation and procedures given in [22] [23] [24].

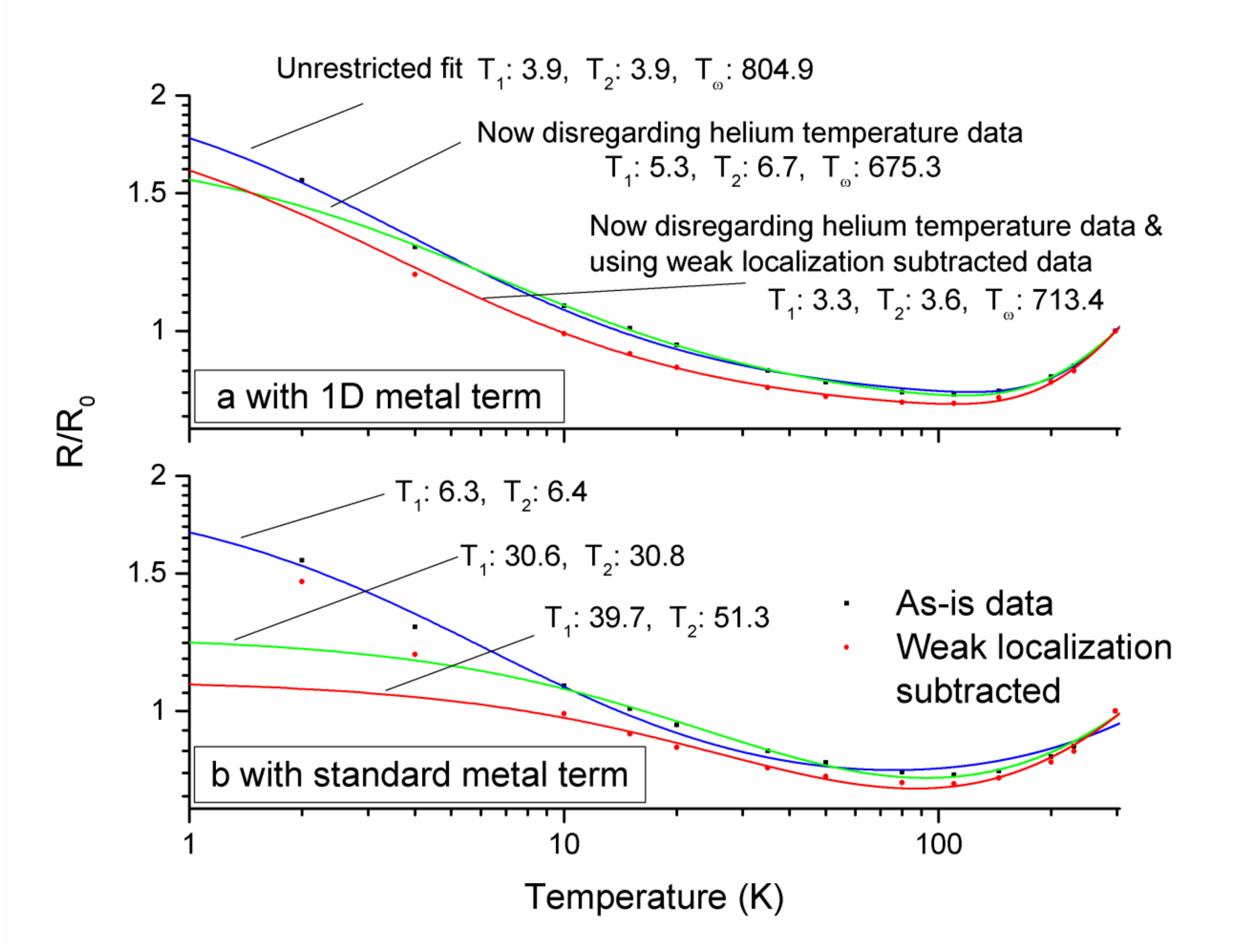

Figure S6-1| Fitting *R* versus *T* to the fluctuation model for the data as is, then excluding data less than 4 K (because of possible EEI interactions), and then excluding the data less than 4 K and excluding the contribution from weak localization. **a**, covers the fitting with an anisotropic, quasi 1D conduction term and **b** is with the standard metallic conduction term. R_0 is the resistance at room temperature.

Supplemental Works Cited

[1] K. Yanagi, H. Udoguchi, S. Sagitani, Y. Oshima, T. Takenobu, H. Kataura, T. Ishida, K. Matsuda and Y. Maniwa, "Transparent mechanisms in metallic and semiconducting single-wall carbon nanotube networks," *ACS Nano,* vol. 4, no. 7, pp. 4027 - 4032, 2010.

[2] J. Vavro, J. M. Kikkawa and J. E. Fischer, "Metal-insulator transistion in doped single-wall carbon nanotubes," *Phys. Rev. B,* vol. 71, p. 155410, 2005.

[3] A. Kurobe and H. Kamimura, "Correlation effects on variable range hopping conduction and the magnetoresistance," *J. Phys. Soc. Jpn.,* vol. 51, pp. 1904 - 1913, 1982.

[4] A. Frydman and Z. Ovadyahu, "Spin and quantum interference in hopping conductivity," *Solid State Commun.,* vol. 94, no. 9, pp. 745 - 749, 1995.

[5] R. Rosenbaum, T. Murphy, E. Palm, S. Hannahs and B. Brandt, "Magnetoresistance of insulating amorphous NixSi(1-x) films exhibiting Mott variable-range hopping laws," *Phys. Rev. B,* vol. 63, p. 094426, 2001.

[6] T. Takano, T. Takenobu and Y. Iwasa, "Enhancement of carrier hopping by doping single walled carbon nanotube films," *J. Phys. Soc. Jpn. ,* vol. 77, p. 124709, 2008.

[7] G. Bergmann, "Weak localization in thin films - a time-of-flight experiment with conduction electrons," *Phys. Rep.,* vol. 107, no. 1, pp. 3 - 58, 1984.

[8] S. N. Song, X. K. Wang, R. P. H. Chang and J. B. Ketterson, "Electronic properties of graphite nanotubules from galvanomagnetic effects," *Phys. Rev. Lett.,* vol. 72, no. 5, pp. 697 - 701, 1994.

[9] R. Bhatia, V. Prasad and R. Menon, "Probing the inter-tube transport in aligned and random multiwall carbon nanotubes," *J. Appl. Phys.,* vol. 109, p. 053713, 2011.

[10] P. A. Lee and T. Ramakrishnan, "Disordered electronic systems," *Rev. Mod. Phys.,* vol. 57, no. 2, pp. 287 - 337, 1985.

[11] V. Bayot, L. Piraux, J.-P. Michenaud, J.-P. Issi, M. Lelaurain and A. Moore, "Two-dimensional weak localization in partially graphitic carbons," *Phys. Rev. B,* vol. 41, no. 17, pp. 11770 - 11779, 1990.

[12] V. Bayot, L. Piraux, J.-P. Michenaud and J.-P. Issi, "Weak localization in pregraphitic carbon fibers," *Phys. Rev. B,* vol. 40, no. 6, pp. 3514 - 3523, 1989.

[13] L. Piraux, "Weak localization and coulomb interaction in graphite intercalation compounds and related materials," *J. Mater. Res,* vol. 5, no. 6, pp. 1285 - 1298, 1990.

[14] P. K. Choudhury, M. Jaiswal and R. Menon, "Magnetoconductance in single-wall carbon nanotubes: Electron-electron interaction and weak localization contributions," *Phys. Rev. B,* vol. 76, p. 235432, 2007.

[15] J. Cai, L. Lu, W. Kong, H. Zhu, C. Zhang, B. Wei, D. Wu and F. Liu, "Pressure-induced transition in magnetoresistance of Single-walled carbon nanotubes," *Phys. Rev. Lett.,* vol. 97, p. 026402, 2006.

[16] G. McIntosh, G. Kim, J. Park, V. Krstic, M. Burghard, S. Jhang, S. Lee, S. Roth and Y. Park, "Orientation dependence of magneto-resistance behaviour in a carbon nanotube rope," *Thin Solid Films,* vol. 417, pp. 67 - 71, 2002.

[17] M. Salvato, M. Lucci, I. Ottaviani, M. Cirillo, S. Orlanducci, F. Toschi and M. Terranova, "Weak localization and dimensional crossover in carbon nanotube systems," *Eur. Phys. J. B,* vol. 85, no. 109, pp. 1 - 5, 2012.

[18] J.-C. Charlier and J.-P. Issi, "Electronic structure and quantum transport in carbon nanotubes," *Appl. Phys. A,* vol. 67, pp. 79 - 87, 1998.

[19] S. Hikami, A. I. Larkin and Y. Nagaoka, "Spin-orbit interaction and magnetoresistance in the two dimensional random system," *Prog. Theor. Phys.,* vol. 63, no. 2, pp. 707 - 710, 1980.

[20] G. Kim, E. Choi, D. Kim, D. Suh and Y. Park, "Magnetoresistance of an entangled single-wall carbon-nanotube network," *Phys. Rev. B.,* vol. 58, no. 24, p. 16064, 1998.

[21] S. Ahn, Y. K. Y. Nam, H. Yoo, J. Park and Y. Park, "Magnetotransport in iodine-doped single-walled carbon nanotubes," *Phys. Rev. B,* vol. 80, p. 165426, 2009.

[22] A. Kaiser, "Electronic transport properties of conducting polymers and carbon nanotubes," *Rep. Prog. Phys.,* vol. 64, pp. 1 - 49, 2001.

[23] A. Kaiser, V. Skakalova and S. Roth, "Modelling conduction in carbon nanotube networks with different thickness, chemical treatment and irradiation," *Physica E,* vol. 40, pp. 2311-2318, 2007.

[24] P. Sheng, "Fluctuation-induced tunneling conduction in disordered materials," *Phys. Rev. B,* vol. 21, pp. 2180 - 2195, 1980.
